# A New Method Based on the von Mises-Fisher Distribution Shows that a Minority of Liver-Localized CD8 T Cells Display Hard-To-Detect Attraction to *Plasmodium*-Infected Hepatocytes

**DOI:** 10.3389/fbinf.2021.770448

**Published:** 2022-01-31

**Authors:** Viktor S. Zenkov, James H. O’Connor, Ian A. Cockburn, Vitaly V. Ganusov

**Affiliations:** ^1^ Electrical Engineering and Computer Science, University of Tennessee, Knoxville, TN, United States; ^2^ Division of Immunology, Inflammation and Infectious Disease, John Curtin School of Medical Research, Australian National University, Canberra, ACT, Australia; ^3^ The Australian National University Medical School, Australian National University, Canberra, ACT, Australia; ^4^ Department of Microbiology, University of Tennessee, Knoxville, TN, United States; ^5^ Department of Mathematics, University of Tennessee, Knoxville, TN, United States

**Keywords:** CD8 T cell, malaria, biased movement, simulated movement, cell movement model, attracted movement

## Abstract

Malaria is a disease caused by *Plasmodium* parasites, resulting in over 200 million infections and 400,000 deaths every year. A critical step of malaria infection is when sporozoites, injected by mosquitoes, travel to the liver and form liver stages. Malaria vaccine candidates which induce large numbers of malaria-specific CD8 T cells in mice are able to eliminate all liver stages, preventing fulminant malaria. However, how CD8 T cells find all parasites in 48 h of the liver stage lifespan is not well understood. Using intravital microscopy of murine livers, we generated unique data on T cell search for malaria liver stages within a few hours after infection. To detect attraction of T cells to an infection site, we used the von Mises-Fisher distribution in 3D, similar to the 2D von Mises distribution previously used in ecology. Our results suggest that the vast majority (70–95%) of malaria-specific and non-specific liver-localized CD8 T cells did not display attraction towards the infection site, suggesting that the search for malaria liver stages occurs randomly. However, a small fraction (15–20%) displayed weak but detectable attraction towards parasites which already had been surrounded by several T cells. We found that speeds and turning angles correlated with attraction, suggesting that understanding mechanisms that determine the speed of T cell movement in the liver may improve the efficacy of future T cell-based vaccines. Stochastic simulations suggest that a small movement bias towards the parasite dramatically reduces the number of CD8 T cells needed to eliminate all malaria liver stages, but to detect such attraction by individual cells requires data from long imaging experiments which are not currently feasible. Importantly, as far as we know this is the first demonstration of how activated/memory CD8 T cells might search for the pathogen in nonlymphoid tissues a few hours after infection. We have also established a framework for how attraction of individual T cells towards a location in 3D can be rigorously evaluated.

## 1 Introduction

Malaria is a disease caused by parasites of the genus *Plasmodium* that kills over 400,000 people every year (WHO, 2018). Mosquitoes carrying malaria sporozoites inject sporozoites when searching for blood (Aleshnick et al., 2020). Sporozoites travel through the bloodstream to the liver, invade hepatocytes, and form liver stages. The liver stage of malaria infection is asymptomatic. The development of the liver stage takes 48 h in mice and 7 days in humans (Murphy et al., 1989; Hermsen et al., 2001; Sturm et al., 2006; Miller et al., 2013). Importantly, vaccines inducing exclusively malaria-specific CD8 T cells are capable of removing all liver stages in mice, thus preventing clinical malaria (Schmidt et al., 2008; Schmidt et al., 2010; Schmidt and Harty, 2011). Intravital imaging experiments showed that soon after infection, activated *Plasmodium*-specific CD8 T cells and CD8 T cells of irrelevant specificity cluster around *Plasmodium*-infected hepatocytes (Cockburn et al., 2013; Kelemen et al., 2019). Mathematical modeling-based analysis of the T cell clustering data suggested that the formation of the clusters is best explained by a model in which the first T cell finds the liver stage randomly and the attraction of other T cells (including T cells with irrelevant specificity) to the parasite increases with the number of T cells per cluster (Kelemen et al., 2019). While an earlier study suggested that there may be attraction of distant T cells to the clustered liver stage (Kelemen et al., 2014), it remains unclear if attraction to the liver stage occurs prior to the formation of T cell clusters around parasites, as well as if attraction is exhibited by all or just a subset of cells.

Based on intravital imaging experiments, in many if not most analyzed cases, T cells appear to move randomly in tissues *in vivo* (Krummel et al., 2016a). Few studies have accurately quantified if motile T cells exhibit biased migration. In part, this is because of the difficulty in quantifying bias in movement patterns of T cells and relating the movement bias to specific structures in the tissue. Previous studies on the movement of naive B cells in B cell zones of the lymph nodes showed a difference in migration towards the boundary of the follicle, or the plane separating light and dark zones of the germinal center reactions (Okada et al., 2005a; Beltman et al., 2011). A more rigorous analysis of movement of virus-specific CD8 T cells in the skin demonstrated attraction of T cells to infection sites (Ariotti et al., 2015). However, these studies employed simple metrics to measure attraction (e.g., percent of cells moving to a particular area or the distance between a cell and the boundary) and in most cases required a comparison group to estimate bias. Also, there was no analysis into if any of these metrics have inherent biases. For example, T cells tend to move with a persistent random walk in which a movement’s direction tends to be similar to the previous movement’s direction (Beltman et al., 2009), which may generate an illusion of a bias towards a specific location. While methods to detect attraction to specific locations have been proposed and studied extensively in ecology (Turchin et al., 1991; Turchin, 1998; Duchesne et al., 2015), ecological movement data are typically 2D. Whether previously proposed methods apply to 3D situations (e.g., for T cells moving in the liver) has not been thoroughly investigated.

At a fundamental level, it is mostly unknown how vaccine-induced T cells search for *in vivo* sites of pathogen replication in peripheral tissues in the hours after infection. This remains an experimental challenge because at early time points, individual pathogens may have too low fluorescent signal to be easily detectable experimentally, and it is difficult to localize memory CD8 T cells near infection sites. To address this fundamental knowledge gap and to understand how CD8 T cells search for the *Plasmodium* liver stages, we performed novel intravital microscopy-based experiments with murine livers in which we tracked positions of liver-localized fluorescently labeled malaria-specific CD8 T cells, CD8 T cells of irrelevant specificity, and malaria liver stages. In our experiments, *Plasmodium* sporozoites expressed GFP at a sufficiently high level so that individual parasites could be followed in the liver minutes to hours after intravenous inoculation (Wang et al., 2020).

To evaluate if T cells display attraction towards infection sites, we first utilized previously-used metrics (and developed a correction to an intuitive metric based on the change in distance between T cells and the parasite), then ultimately developed a new metric based on applying the von Mises-Fisher (vMF) distribution to angles to a parasite. This new metric is more sensitive (requires less data to detect deviations from random movement) than previously used metrics. Similar to how we found bias in the existing distance metric, we found that our vMF distribution-based metric can be slightly biased for T cells with a correlated/persistent random walk, but we use simulations to approximately correct that bias. Our results suggest that CD8 T cells do not display attraction towards the infection site, with one exception: when a liver stage already possesses a CD8 T cell cluster, a minority of T cells do display detectable attraction to this infection site. Stochastic simulations based on the vMF distribution suggest that the detection of weak attraction of individual cells towards the infection site requires amounts of data that are difficult-to-impossible to collect with current intravital imaging protocols. Our work establishes a rigorous framework for evaluating the attraction of moving cells towards a particular location, and begins to explain how malaria-specific CD8 T cells navigate in the presence of malaria infection.

## 2 Materials and Methods

### 2.1 Experimental Design

Our data consists of 3D positions over time of CD8 T cells specific for malaria sporozoites; malaria liver stages; and in some cases, control CD8 T cells specific to irrelevant antigens. We analyzed datasets from five sets of experiments: three datasets generated for this analysis and two from a published study (Cockburn et al., 2013). Experimental parameters are described below. For all mice, a lateral incision was made over the left lobe of the liver and the liver was exposed; then the mouse was transfered to the microscope system for imaging. Comprehensive experimental details are provided in the Supplemental information.

For the new “unclustered/small clustered” (dataset #1) and “large clustered” (dataset #2) datasets, mice were infected with GFP-expressing *Plasmodium berghei* (Pb-CS^5M^) sporozoites, which carry the SIINFEKL epitope from OVA (Cockburn et al., 2014; McNamara et al., 2017). Activated CD8 T cells, specific to SIINFEKL epitope (OT1), were generated *in vitro* by co-culture of naive TCR transgenic CD8 T cells with SIINFEKL peptide as described previously (McNamara et al., 2017). As a control, we used TCR-transgenic CD8 T cells, specific to GP33 epitope from LCMV (P14) that were activated similarly to the OT1 cells. Our B6 mice received 5 × 10^6^ activated Pb-specific (OT1) or LCMV-specific (P14) CD8 T cells and then 1.5–2 h later, were infected with 10^5^ Pb-CS^5M^ sporozoites (Supplementary Figure S1). We performed imaging between 30 min and 2 h after sporozoite infection using a two-photon microscope (McNamara et al., 2017). The unclustered/small clustered dataset experiments featured no or few T cells located within 40 *μ*m from the parasite at the beginning of the experiment, and the large clustered dataset experiments featured several T cells located within 40 *μ*m from the parasite (i.e., there was a T cell cluster at the beginning of the experiment (Cockburn et al., 2013)). We used a 40 *μ*m radius to distinguish closeness because this value has been used to represent the average radius of a standard murine hepatocyte when roughly modeled as a sphere (Kelemen et al., 2019). The unclustered/small clustered dataset contains 3D coordinates over 3 h (with timesteps of 1.5 or 2 min between recorded stacks of images) of Pb-specific (OT1) and LCMV-specific (P14) CD8 T cells in 4 mice (1 parasite per mouse, Supplementary Figure S1, S2). The large clustered dataset contains 3D coordinates over 3 h (with timesteps of 1 or 2 min between recorded stacks of images) of Pb-specific and LCMV-specific CD8 T cells for 3 mice (1 parasite per mouse). As a control, we also performed an experiment in which naive B6 mice received 5 × 10^6^ activated Pb-specific (OT1) cells but no sporozoites (i.e., no infection was given), and livers of the mice were imaged 1.5–2 h after T cell transfer. This “No Parasite” dataset (dataset #3) contains 3D coordinates over 30 min (with timesteps of 30 s between recorded stacks of images) for 1 mouse.

We also analyzed datasets from our previous study (Cockburn et al., 2013). To generate the “Paris” dataset (dataset #4), the following experimental set-up was used. Activated CD8 T cells specific for *Plasmodium yoelii* (Py) sporozoites (PyTCR) were generated *in vivo* by infecting Balb/c mice with the Vaccinia virus expressing the circumsporozoite (CS) protein from Py (Cockburn et al., 2013). Balb/c mice were first infected with Py sporozoites, then 20 h later, activated Py-specific (PyTCR) CD8 T cells (5 × 10^6^ per mouse) were transferred to the infected mice intravenously. Imaging of the livers of these infected mice was performed 6 h after the T cell transfer (Cockburn et al., 2013). To generate the “co-clustered” dataset (dataset #5), PyTCR cells were activated as described above. In addition, CD8 T cells specific to chicken ovalbumin (OT1) were activated by infecting B6 mice with the Vaccinia virus expressing OVA. CB6 mice (F1 progeny of B6 and Balb/c mice) were infected with 10^5^ Py sporozoites; 20 h later activated Py-specific (PyTCR, 5 × 10^6^ per mouse) and OVA-specific (OT1, 5 × 10^6^ per mouse) CD8 T cells were transferred into infected mice. Six hours later, livers of these mice were imaged. In both experiments, we performed intravital imaging using spinning-disk confocal microscopy. The Paris dataset contains 3D coordinates over 5 h (with timesteps of 1, 2, or 4 min between recorded stacks of images) of Py-specific CD8 T cells for 26 parasites in 4 mice. The co-clustered dataset contains 3D coordinates over 40 min (with timesteps of 2 min between recorded stacks of images) of Py-specific and OVA-specific CD8 T cells for 1 parasite in 1 mouse. Note that the terms “parasite” and “infection site” are used interchangeably in much of the paper, as well as “liver stage” when discussing the malaria lifecycle.

All 3D coordinate data are available as a Supplemental information to this paper. We also provide a Mathematica-based script that allows one to estimate the bias of moving agents to a point or a plane based on our newly developed vMF distribution-based metric, at https://github.com/viktorZenkov/measuringAttraction/tree/master/Measuring. In addition, we provide 2 Imaris files that contain the movies from one small clustered/unclustered experiment and one large clustered experiment, featuring the “Spots” tracks of all T cells and the parasite in the imaging volumes (https://doi.org/10.5281/zenodo.5715658).

### 2.2 Metrics

To measure T cell bias (attraction or repulsion) towards the parasite, we define the following 4 metrics (Supplementary Figure S3).

1. Angle metric (metric 1). For every movement of a cell, we calculate the angle between the cell’s movement angle and the angle to the parasite (Supplementary Figure S3A). An acute angle corresponds to the T cell “getting closer”, and an obtuse angle corresponds to the T cell “getting farther.” For an unbiased cell making *n* movements, the choices of closer/farther for the movements are given by a binomial distribution with *p* = 0.5. This metric has been used extensively in ecology (Turchin, 1998).
2. Distance metric (metric 2). For every movement of a cell, we calculate *D*0, the change in the distance between the cell’s current position and the parasite, and *D*1, the distance between the cell’s next position and the parasite, with the change in distance designated by *r* = *D*0 − *D*1 (Supplementary Figure S3B). A negative change in distance corresponds to “getting closer” and positive corresponds to “getting farther”. For an unbiased cell making *n* movements, the choices of closer/farther for the movements are given by a Poisson Binomial distribution with *p* = 0.5 − *r*/4*x*, where *r* is the cell’s movement length and *x* = *D*0 is the initial distance between the cell and the parasite, or *p* = 0 when *r* > *x* (Supplementary Figure S4) (Hong, 2013). This choice of null distribution is a critical improvement that we made which is necessary for this test to detect attraction at its fullest strength, which is explained further in the Supplemental information.
3. Angle distribution metric (metric 3). For every movement of a cell, we calculate the angle between the cell’s movement vector and the vector to the parasite and compare all angles with the von Mises-Fisher (vMF) distribution (Supplementary Figure S3C and Eq. 1) (Fisher, 1987; Mardia, 2000). By fitting the vMF distribution to the angle data, we calculate a concentration parameter *κ* which indicates the strength of a cell’s attraction towards the infection site, with *κ* > 0 indicating attraction and *κ* < 0 indicating repulsion. A 2D version of a similar, von Mises distribution-based metric, has been used in ecology (Duchesne et al., 2015).
4. Average angle metric (metric 4). For every movement of a cell, we calculate the angle between the cell’s movement vector and the vector to the parasite (Castellino et al., 2006). We then use a Student’s t test to compare the mean of the angles to the expected average angle of 90° with no attraction/repulsion (Castellino et al., 2006; Ariotti et al., 2015).

More information on the statistical tests using these metrics and the choices of null distributions, including thorough mathematical overviews demonstrating the validity of these tests, is provided in the Supplemental information.

### 2.3 von Mises-Fisher Distribution

To quantify the degree of bias (or absence thereof) we introduce the von Mises-Fisher (vMF) distribution of angles towards the parasite (Jakob, 2012). The vMF distribution describes a probability distribution on an *n*-dimensional (we use *n* = 3) sphere given a direction vector and a concentration parameter *κ*. Sampling from the distribution gives a vector chosen pseudorandomly with a bias toward the given direction whose strength depends on *κ*. When *κ* → 0, the vMF distribution approaches a uniform distribution; *κ* > 0 indicates positive bias (attraction); and *κ* < 0 indicates negative bias (repulsion, Supplementary Figure S5). We reduce the vMF distribution from a vector to a single angle between the output vector and the given vector - this angle *ϕ* corresponds to our angle metric. The probability density function of the angles of the vMF distribution with respect to the point of attraction is

$$P\left(\phi |\kappa \right)=\frac{\kappa \sin\left(\phi \right)e^{\kappa \cos\left(\phi \right)}}{2\sinh\left(\kappa \right)},\;0\leq \phi \leq \pi ,$$ (1)

where *ϕ* is the angle between the vector to the attraction point and the cell’s movement vector. More intuitive parameters such as the fraction of acute angles *f*_*a*_ or the average angle towards infection $\bar{\phi }$ can be calculated as well (see Supplementary Equation S4 in Supplemental information). Multiple biases, such as towards previous movement vectors or towards the infection site, can be naturally incorporated into a model using multiple vMF distributions (see Supplemental information).

To estimate the concentration parameter *κ*_*a*_ of a set of movement angles, we used the maximum likelihood approach (Supplementary Equation S5); more specifically, we used the Maximize function in Mathematica to estimate *κ*_*a*_. To evaluate if the estimated concentration parameter is statistically different from 0, we used a log-likelihood test by comparing the negative log-likelihood of the best fit and the negative log-likelihood with *κ*_*a*_ → 0. Details of how the vMF distribution can be used to simulate random walks with biases, a minor correction to the test similar to the improvement we made to the distance metric, and an analysis illustrating that the vMF distribution-based metric is the most powerful out of our tested metrics to detect attraction, are given in the Supplemental information (e.g., see Supplementary Figures S6, S7).

### 2.4 Simulations

To understand various aspects of T cell movement with respect to a parasite, we performed stochastic simulations using Mathematica 12.0. In our simulations, cell movements are characterized by a distance traveled per time step and a direction of cell movement (with respect to the previous movement and/or to the parasite). Movement lengths are chosen from a Generalized Pareto (Pareto type IV) distribution, and the direction of the movement is chosen from a vMF distribution with a given concentration parameter *κ*. Additionally, we developed a new method to simulate attracted cell movement using a variation of the Ornstein-Uhlenbeck process, one of the main methods used in biophysics to simulate correlated random walks (Jerison and Quake, 2020). Details of our simulation methods are provided in the Supplemental information.

## 3 Results

### 3.1 Only a Small Proportion of Activated Liver-Localized CD8 T Cells Display Attraction Towards Malaria Liver Stages

**FIGURE 1:**
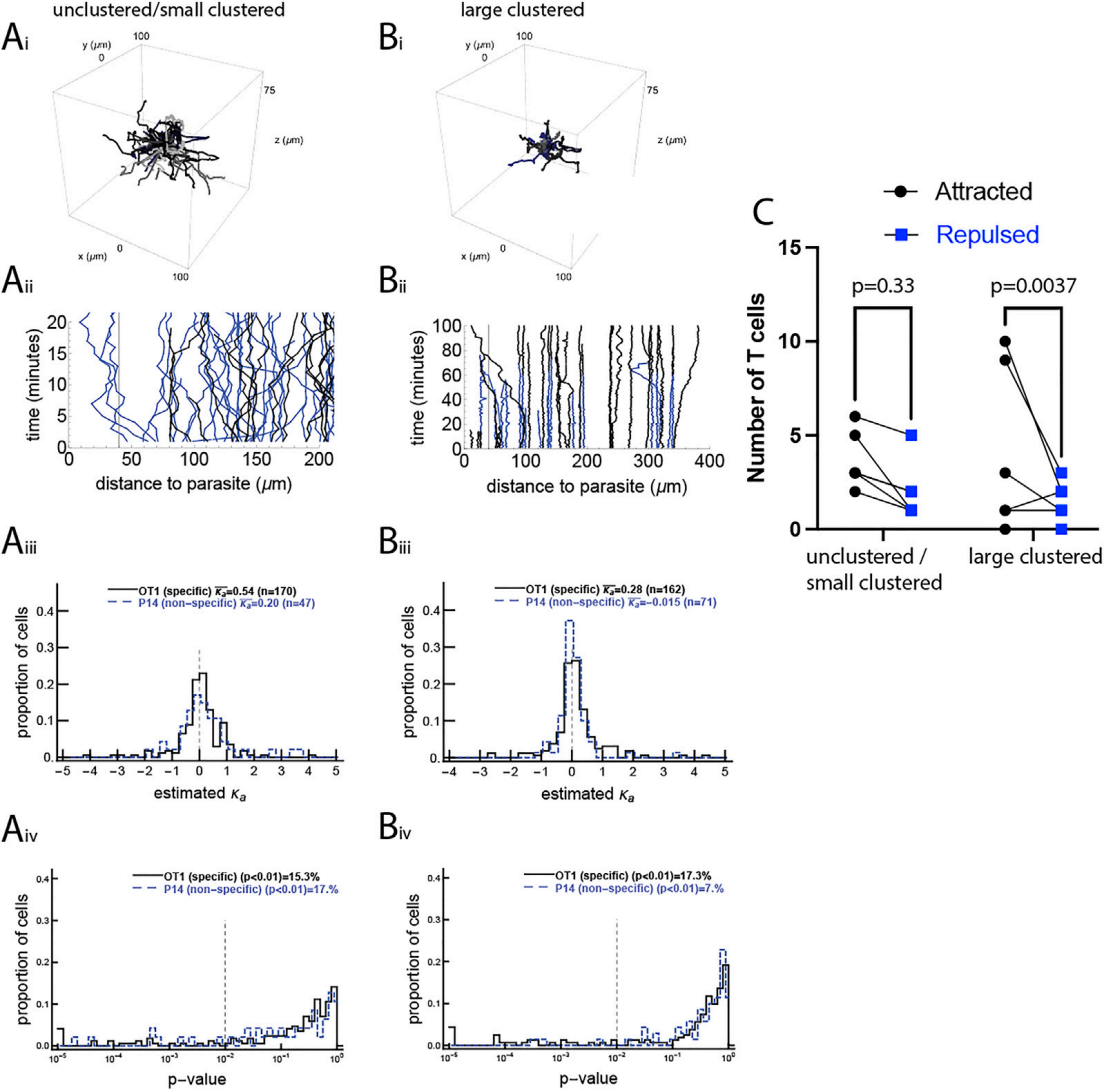
A minority of activated CD8 T cells display movement bias to the malaria liver stage. We performed four sets of experiments in which the movement of CD8 T cells with respect to the location of the malaria liver stage was recorded using intravital microscopy (see Materials and Methods), and performed tests on this movement data. In the unclustered/small clustered dataset, the movement of malaria-specific (OT1) CD8 T cells and T cells of irrelevant specificity (P14) was recorded around a single liver stage of Pb when no or few T cells were near the parasite (dataset #1, panels **(A)**, 4 movies in total). In the large clustered dataset, the movement of malaria-specific (OT1) CD8 T cells and T cells of irrelevant specificity (P14) was recorded around a single liver stage of Pb when several T cells were already near the parasite (dataset #2, panels **(B)**, 3 movies in total). Panels i show the T cell tracks from one of each set of experiments (the second infection site of the unclustered/small clustered data and the first infection site of the large clustered data) and panels ii show the distance from each T cell to the liver stage over time for the same infection sites. For each T cell we calculated the bias of T cell movement towards the parasite by estimating the concentration parameter *κ*_*a*_ from the vMF distribution (see Eq. 1 and Supplementary Figure S3) using the maximum likelihood method. Panels iii show the distribution of estimated *κ*_*a*_s and panels iv show *p*-values from the likelihood ratio test for the vMF distribution with $\kappa_{a}\to \kappa_{a}^{0}$ , where $\kappa_{a}^{0}$ is the threshold value from the null distribution (see text for more detail of how $\kappa_{a}^{0}$ was calculated). The average concentration for all cells is shown on individual panels in iii, and the percent of T cells with a statistically significant bias to the parasite (with *p* ≤ 0.01 from our log-likelihood test) is shown in panels iv. Biased cells include both attracted (*κ*_*a*_ > 0) and repulsed (*κ*_*a*_ < 0) cells. In the unclustered/small clustered dataset, we detected 16 OT1 and 5 P14 cells as attracted and 10 OT1 and 3 P14 as repulsed. In the large clustered dataset, we detected 22 OT1 and 2 P14 cells as attracted and 6 OT1 and 3 P14 as repulsed. The number of OT1 cells detected as attracted was significant for the large clustered dataset and not significant for the unclustered/small clustered dataset **(C)**. Results of the analyses for two other datasets are given in the Supplemental information (Supplementary Figure S8).

To determine if liver-localized CD8 T cells display attraction towards malaria liver stages, we performed novel experiments in which we tracked positions of malaria-specific CD8 T cells, CD8 T cells with irrelevant specificity, and malaria liver stages over time in murine livers using intravital microscopy (see Materials and Methods for more details). We attempted to design our experiments to detect a “first contact” event in which the first T cell locates the infection site, and such an event did occur in one experiment (described in section 3.3). In all other experiments, by the starting time of imaging, a small (1–2) or large number (5–7) of T cells had already formed a cluster around the liver stage (Supplementary Figure S9). In general, imaging experiments performed later after the sporozoite infection resulted in larger clusters (results not shown). Notably, T cells displayed different movement characteristics depending on whether there were no/few or many cells in the cluster (Figure 1i–ii). Specifically, in unclustered/small clustered data, cell speeds were 2.42 ± 2.62 and 2.63 ± 3.03 *μ*m/min for OT1 and P14 cells, respectively (mean ± standard deviation, Supplementary Figure S10). In the large clustered data, both cell types were significantly slower, with speeds of OT1 and P14 cells being 1.55 ± 1.66 and 1.59 ± 1.30 *μ*m/min, respectively (Mann-Whitney test, *p* < 0.001). In the presence of large clusters, T cells were likely to have a larger arrest coefficient (fraction of cell movements with a speed below 1 *μ*m/min): 0.29 and 0.27 for OT1 and P14 cells, respectively, in unclustered/small cluster data vs 0.47 for both cell types in large cluster data. Additional analysis based on the meandering index (the distance between the first and last recorded positions divided by the total length of the path) and turning angles for T cells showed that all cells tend to turn (Supplementary Figure S11) suggesting an active search for an infection.

To understand how T cells search for the infection site, we first pooled all track data from a given dataset into one set and determined if T cells display attraction towards the infection by fitting a vMF distribution to the data and estimating the concentration parameter *κ*_*a*_ (see Materials and Methods for more detail). We found that T cells displayed a statistically significant but weak attraction towards the parasite (*κ*_*a*_ = 0.077 (LRT, *p* = 3.8 × 10^–4^) and *κ*_*a*_ = 0.085 (LRT, *p* = 3.9, ×, 10^–5^) for the unclustered/small clustered and large clustered datasets, respectively, Figure 1); the concentration parameter *κ*_*a*_ = 0.077 corresponds to only 52% of movements being towards the parasite. There was stronger attraction of *Plasmodium*-specific CD8 T cells towards the infection site detected in the other two datasets with infections (*κ*_*a*_ = 0.19 (*p* = 0.003) and *κ*_*a*_ = 0.51 (*p* = 0.001), in Supplementary Figures S8, S12).

To investigate if weak attraction towards the parasite comes from all cells exhibiting weak attraction or a few cells exhibiting strong attraction, we calculated *κ*_*a*_ for individual cells. There was a broad distribution of *κ*_*a*_ for individual cells, but most of these values were not statistically different from a random value, with the average of these values indicating attraction (Figure 1iii-iv and Supplementary Figure S8iii–iv). It should be noted that because the vMF distribution-based metric is inherently slightly biased for T cells moving with a correlated random walk, for every T cell track we calculated the null hypothesis concentration parameter $\kappa_{a}^{0}$ that would be expected given the cell’s initial position (with respect to the parasite and imaging volume), turning angle distribution, and movement lengths, and statistically compared the true *κ*_*a*_ to the null hypothesis value $\kappa_{a}^{0}$ (results not shown). In most cases, this calculated null hypothesis $\kappa_{a}^{0}$ was not very different from zero. Notably, only OT1 cells (specific for malaria liver stages) in the large clustered data displayed statistically significant attraction towards the infection site ( ${\bar{\kappa }}_{a}=0.28$ ; *p* = 0.0065, signed rank test), while P14 cells in the large clustered data and both OT1 and P14 cells in the unclustered/small clustered data did not display significant attraction (*p* > 0.05, signed rank test). Similarly, only malaria-specific T cells (PyTCR) displayed attraction (or weak attraction) for the two other datasets ( ${\bar{\kappa }}_{a}=0.55$ ; *p* = 0.0024 in the co-clustered dataset and ${\bar{\kappa }}_{a}=0.76$ ; *p* = 0.059 for the Paris dataset), while non-specific cells (OT1 in the co-clustered dataset) were not attracted to the infection site ( ${\bar{\kappa }}_{a}=0.41$ ; *p* = 0.13, Supplementary Figure S8).

We found that about 15% of both malaria-specific and non-specific T cells displayed bias to the parasite (*p* < 0.01, Figure 1iv). Interestingly, while the fraction of *Plasmodium*-specific T cells (OT1) displaying bias toward the infection was similar between the unclustered/small clustered and large clustered datasets (15 vs. 17%, respectively), there were more P14 T cells (specific to LCMV) that displayed bias to the parasite in the unclustered/small clustered data than in the large clustered data (17 versus 7%, Figure 1iv), suggesting that some “biased” T cells in the unclustered/small clustered dataset may be an artifact of the statistical analysis. Indeed, we found similar fractions of T cells detected as attracted to or repulsed from the parasite in all cases except for the OT1 cells in the large clustered data (22/28, binomial test *p* = 0.004), suggesting that only during larger clustering is there a detectable bias in (a minority of) malaria-specific CD8 T cells towards the infection site (Figure 1C).

### 3.2 Detecting Bias to the Infection Is Correlated With Higher Cell Speed and Persistence of Movement

**FIGURE 2:**
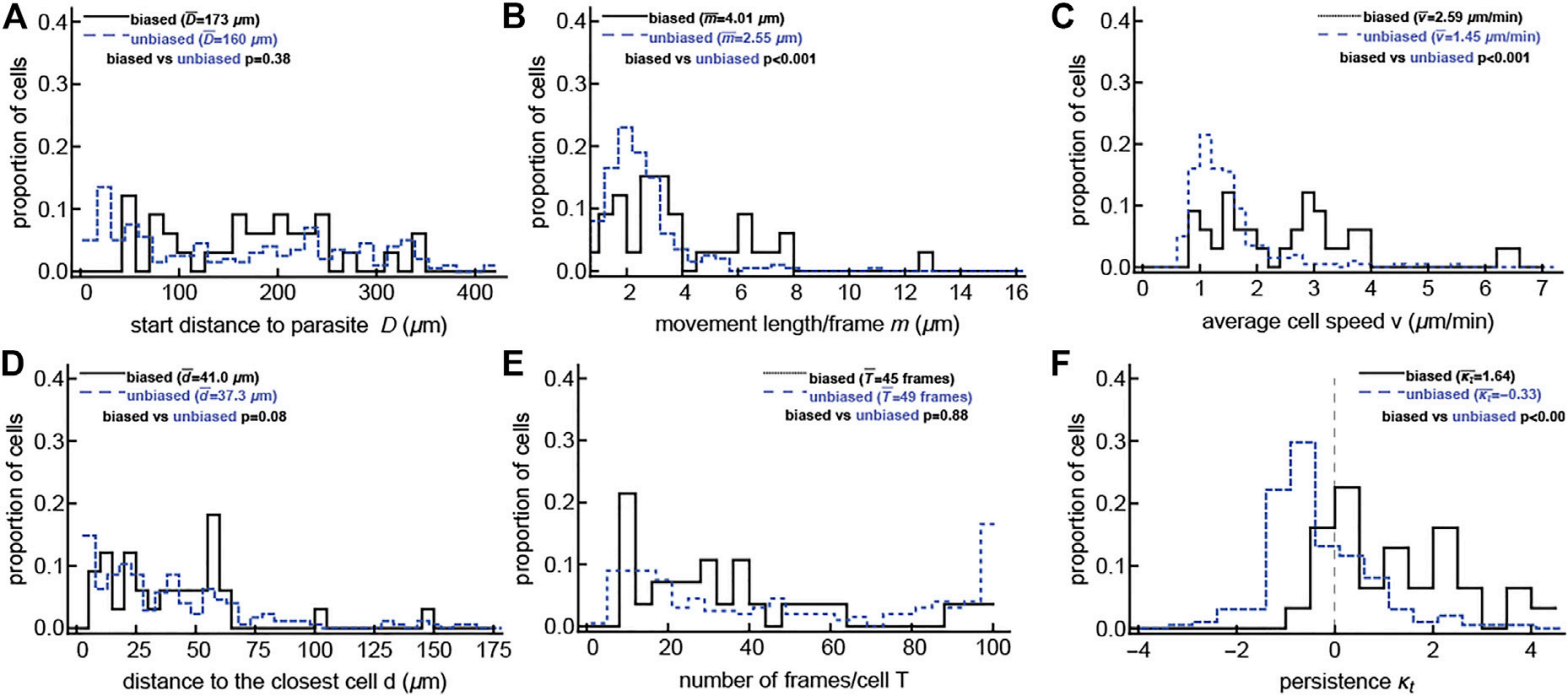
T cells detected as biased are correlated with faster T cells and T cells with smaller turning angles. For the large clustered dataset, in which some T cells have already located the parasite (shown previously in Figure 1B), we calculated multiple characteristics for cells that display no bias to the parasite’s location (“unbiased” cells) and cells that do display bias to the parasite’s position (“biased” cells, i.e., attracted or repulsed). These characteristics include: starting distances *D* **(A)**, movement lengths per frame *m* **(B)**, average speed per T cell *v* **(C)**, distance to the closest T cell *d* **(D)**, the number of recorded positions *T* **(E)**, and the estimated concentration of the vMF for the turning angles *κ*_*t*_ **(F)**. Comparisons were done using the Mann-Whitney test and the *p*-values for the comparisons are shown on individual panels. The unbiased results are offset slightly to not directly overlap with the biased results for ease of viewing. Analyses when the data were divided into attracted, repulsed, or unbiased T cells are shown in Supplementary Figure S13, and analysis of the data with no/small clusters is shown in Supplementary Figure S15. The majority of biased T cells in this dataset display attraction towards the infection (24/33, binomial test *p* = 0.014).

Our analyses suggest that the majority of CD8 T cells searching for malaria liver stages perform such a search without displaying a detectable bias towards the infection site. However, some malaria-specific CD8 T cells do display a detectable bias towards the infection site when there are already some T cells near the parasite. We next sought to determine which T cell characteristics may be correlated with bias towards the infection. To increase the power of the analysis, we pooled the data for malaria-specific CD8 T cells and CD8 T cells of irrelevant specificity. Previously it was suggested that the distance between an infection site and the T cell may determine the strength of attraction (Ariotti et al., 2015). However, we found that the detected degree of attraction did not correlate with the starting distance between T cells and the infection (Figure 2A and Supplementary Figure S13A). The distance to the closest T cell or the overall time per track also did not correlate with attraction (Figures 2D,E and Supplementary Figure S13D,E), while the average movement per time step, cell velocity, and walk persistence (determined by the concentration parameter *κ*_*t*_) strongly correlated with the degree of attraction (Figure 2 and Supplementary Figure S13), suggesting that rapidly moving T cells are more likely to display bias towards the infection site. We also found that for the unclustered/small clustered data, velocity and small turning angles (the latter of which are represented by a large concentration parameter *κ*_*t*_) are correlated with cells displaying bias toward the infection site (Supplementary Figures S14, S15). This may be a statistical artifact because cells moving in a straighter path may randomly have their trajectory aim toward or away from the parasite, which would cause the cells to be detected incorrectly as attracted if the trajectory is toward the parasite and as repulsed if the trajectory is away from the parasite. Therefore faster and more persistent cells may be naturally inclined to be detected as biased toward the cell, but only as a result of chance.

### 3.3 The Amount of Detected Attraction Does Not Noticeably Change Immediately Upon Formation of a Cluster

**FIGURE 3:**
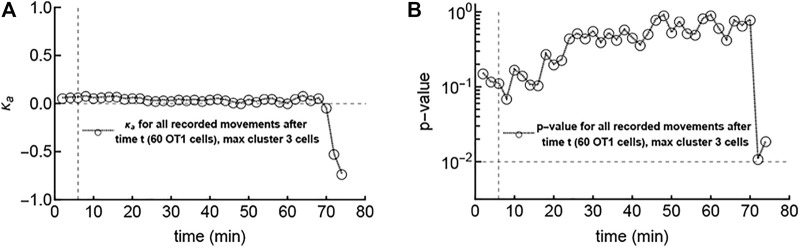
The first scout T cell locating the infection site does not impact the movement bias of other T cells towards the infection within an hour. In this “first contact” experiment (Supplementary Figure S9Bii), no T cells were close to the parasite at the start of imaging, and the first cell found the parasite in 6 minutes (marked with a vertical dashed line in both panels). After this cell, two other cells reached the parasite within a few minutes, but no more cells (out of a total of 60 cells in the video) reached the parasite within an hour (and in fact, one of the three left the parasite). We calculated the concentration parameter *κ*_*a*_ for all T cell tracks combined after different times **(A)** and the corresponding *p*-value from the likelihood ratio test (LRT) indicating deviation of *κ*_*a*_ from 0 **(B)**. The horizontal line in **panel B** denotes a *p*-value of 0.01.

Our results show a greater proportion of attraction toward parasites in data with large CD8 T cell clusters. This potentially suggests that the first cells to reach the parasite may do so randomly, and then other cells begin to show bias after the environment around the parasite has been changed by the first scout cells. In one of our experiments we did observe such a “first contact” event where, at the start of imaging, no T cells were near the parasite, but then 1 cell out of 61 in the imaging volume reached the parasite, and then 2 more cells entered the cluster and 1 cell left (Figure 3 and Supplementary Figure S9Aii). We then analyzed whether those T cells, which were not in the cluster after the first scout T cell located the parasite, displayed bias towards the infection. We pooled the angles of T cell tracks after different times in the experiment and calculated the overall attraction towards the infection, characterized by the concentration parameter *κ*_*a*_ (Figure 3A) and the corresponding *p*-value from the LRT (Figure 3B). Interestingly, the estimated *κ*_*a*_ for angles after an hour after the first scout T cell found the infection site were still not significantly different from zero (Figure 3). A statistically significant repulsion (*κ*_*a*_ < 0) after later time points is most likely the result of noise because of very limited data after late time points. Our analysis suggests that if the first T cell that located the parasite changes the environment, such a change takes longer than ∼50 min to be detected by other moving T cells.

### 3.4 Detecting Weak Attraction Is Difficult With Current Experimental Setups

**FIGURE 4:**
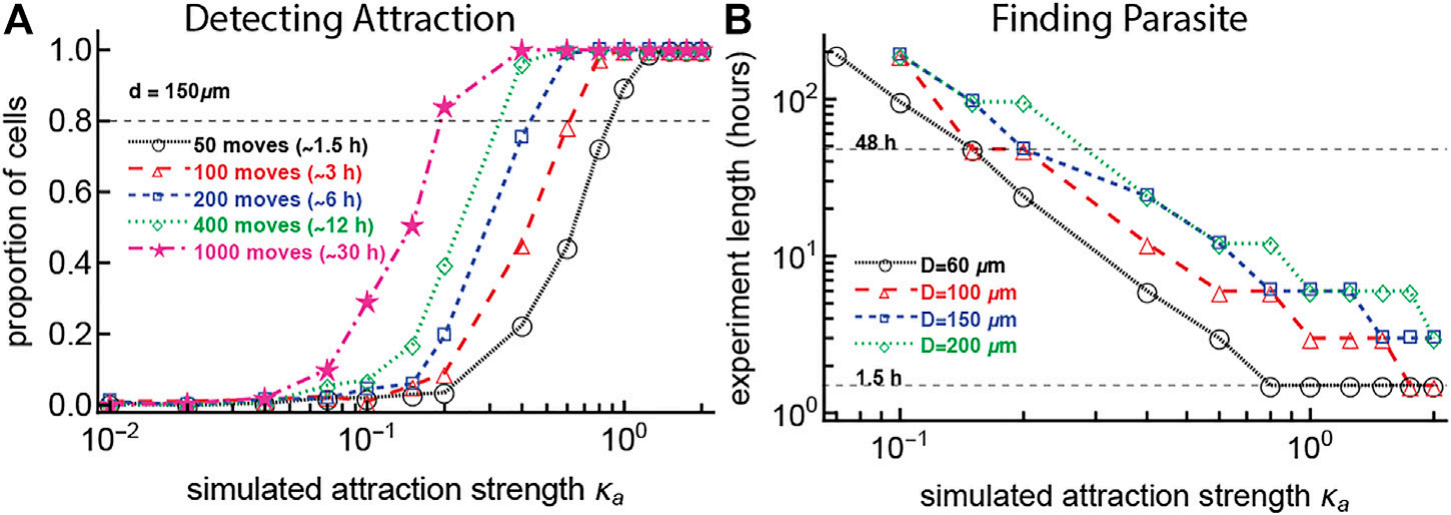
To detect weak T cell attraction to the parasite or to observe a T cell finding the parasite requires a prohibitively long imaging duration. In panel **(A)** we simulated T cell movement with some degree of attraction to the parasite defined by the concentration *κ*_*a*_ and starting at a distance *D* = 150 *μ*m. For 1,000 simulated T cells, we varied the number of T cell movements (which changes the duration of the imaging movie). For every track we then determined the probability that the movement track would exhibit attraction (we estimated *κ*_*a*_ from the simulated tracks using maximum likelihood and used the likelihood ratio test to calculate if the estimated value was significantly different from zero, i.e., from random). In panel **(B)** we performed simulations with 1,000 cells for values of *κ*_*a*_ between 0 and 2, with starting distances of 60, 100, 150, and 200 *μ*m from the parasite and T cell movement lengths chosen from a generalized Pareto distribution (Supplementary Equation S8) with pseudorandomly chosen turning angles. We calculated the time it took for the T cell to reach the parasite (reach a distance of 40 *μ*m from the parasite). A typical imaging duration ( ∼1.5 h ) and the time it takes for liver stages to develop (48 h) are indicated by dashed horizontal lines in panel **(B)**.

In our analyses so far we found that, with some exceptions, the vast majority of liver-localized CD8 T cells search for the malaria liver stages randomly, with little evidence of bias towards the infection site. Yet, we know that with sufficient numbers of liver-localized CD8 T cells, all liver stages will be eliminated within 48 h (Schmidt et al., 2008; Fernandez-Ruiz et al., 2016). We reasoned that while our liver imaging experiments were sufficiently long ( ∼1.5−2 hours ) to detect attraction in some cases, it may be possible that they were still too short to detect any weak attraction displayed by individual T cells. Therefore, we performed several sets of simulations to determine the length of experiments that would be required to detect a given degree of T cell attraction towards the infection site (Figure 4).

We simulated movement of 1,000 cells for combinations of varying strengths of attraction towards the infection site (*κ*_*a*_) and numbers of cell movements (choosing numbers of movements with an 2 min timestep, imitating the real data), assuming that cells start their search at 150 *μ*m from the parasite. For each combination of parameters, we determined the power to detect attraction as the proportion of cells which are detected as attracted based on the angle distribution (vMF distribution-based) metric. For our typical experiment with a length of 1.5 h (about 50 movements of T cells), the attraction strength *κ*_*a*_ must be more than 1.0 for the bias of T cell movement towards infection to always be detected (Figure 4A). Furthermore, to detect weaker attraction (*κ*_*a*_ = 0.2), 30 h of imaging experiments (or 1,000 movements per cell) would be needed, which is not currently possible due to ethical and experimental constraints. The need for 30 h of data suggests that our failure to detect weak attraction of T cells towards the infection site may be due to limited (but the best currently possible) data.

We wondered how the time to find the parasite may depend on the starting distance from the T cell to the infection site and the degree of the T cell attraction towards the infection site. To answer this, we performed another set of simulations (with 1,000 cells for each combination of parameters) by varying the starting distance between the T cells and the parasite and the degree of T cell attraction to the parasite (*κ*_*a*_). We then calculated the time when a cell found the parasite (reaches within 40 *μ*m from the parasite) with 80% probability. A high degree of attraction (*κ*_*a*_ ∼ 1) and short distance is required for T cells to find the parasite within 1.5 h, and to reach the parasite in 48 h still requires moderate attraction (*κ*_*a*_ = 0.3, Figure 4B). For a T cell with *κ*_*a*_ = 1.0 and a starting distance of 150 *μ*m, the experiment must last for 6 h for the T cell to reach the parasite; currently we are not able to perform experiments with live mice for this length of time. These findings suggest that there may be something special about these parasites that had T cell clusters form around them early after the infection. For example, such parasites may be entering the liver where there are already some T cells nearby, perhaps indicating that *Plasmodium* infection of the liver and T cell localization in the liver do not occur randomly. Furthermore, analyses also suggest that some level of weak attraction is needed to explain T cells finding the parasite in 48 h after infection.

### 3.5 Only a Small Fraction of T Cells Display Bias Towards the Infection Site

**FIGURE 5:**
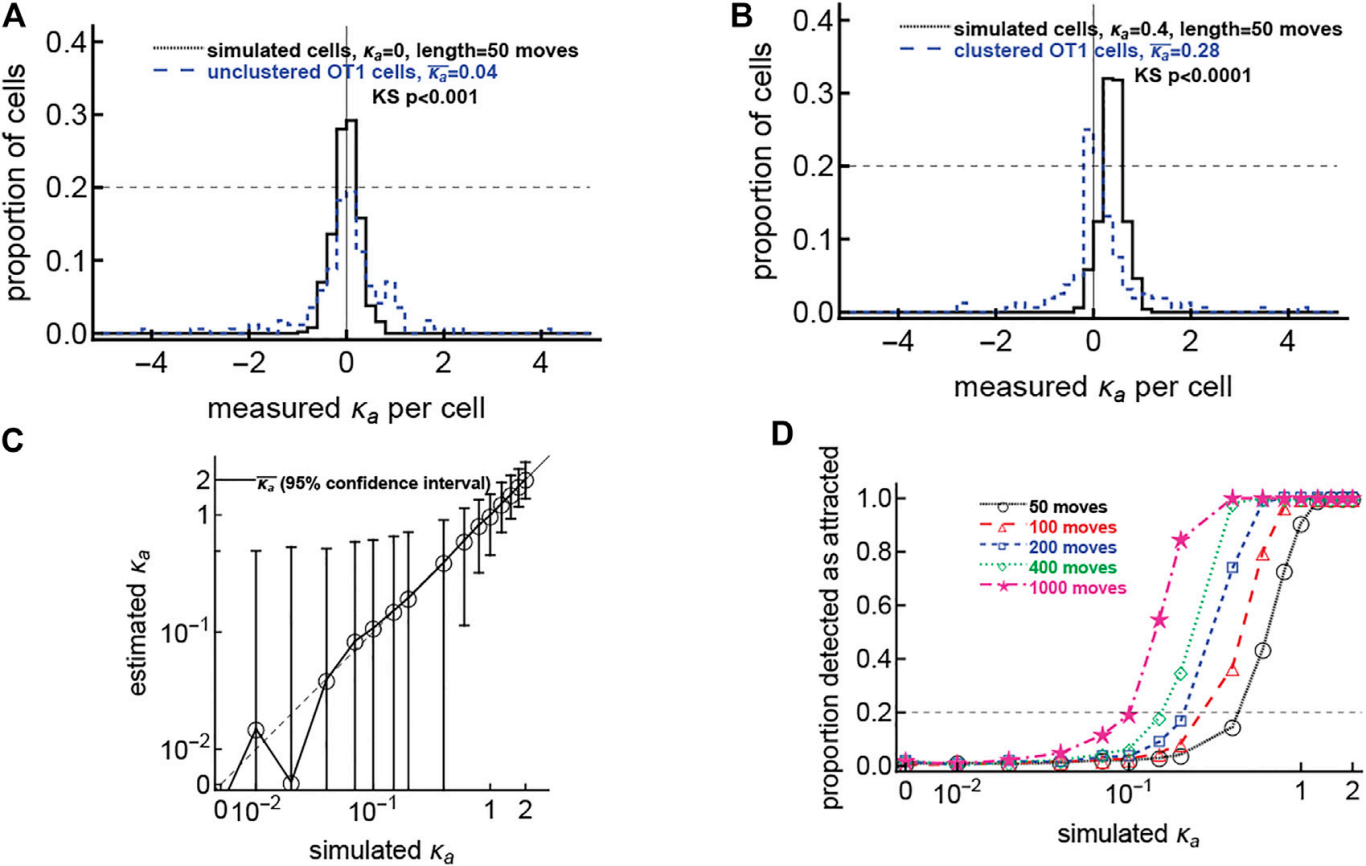
The hypothesis that all cells have weak attraction is not supported by the data. We simulated CD8 T cells searching for a malaria liver stage assuming variable levels of T cell attraction to the parasite, defined by the concentration parameter *κ*_*a*_ of the vMF distribution and with different number of movements (from 50 to 1,000 movements/cell). For each cell we calculated the observed attraction towards the infection site using the vMF distribution (Eq. 1). In panels **(A,B)** we overlay distributions of detected *κ*_*a*_ from simulated and actual datasets. Panel **(A)** shows data for unclustered cells and simulated cells with no inherent attraction (*κ*_*a*_ = 0.0001), while panel **(B)** shows data for clustered cells and simulated cells with a minor attraction (*κ*_*a*_ = 0.1). *p* values using the Kolmogorov-Smirnov (KS) test to compare the experimental and theoretical distributions are shown on the panels. **Panel C** shows the mean estimated *κ*_*a*_ from 500 simulated cells for the simulations with 50 moves and each original *κ*_*a*_; the dashed line denotes the curve *y* = *x*; and error bars denote 95% confidence intervals on the estimated *κ*_*a*_. **Panel D** shows the proportion of cells detected as attracted for each combination of simulated *κ*_*a*_ and number of movements; the horizontal dashed line denotes 20% of cells detected as attracted as we have observed in our experimental data. In simulations, all cells start 150 *μ*m from the parasite. To simulate a T cell walk we assumed that T cell movement lengths follow a generalized Pareto distribution (Supplementary Equation S8) with pseudorandomly chosen turning angles. Simulations were done for 500 cells for each value of *κ*_*a*_ and experiment length.

In our clustered data we found that only about 20*%* of T cells display statistically significant bias in movement towards the infection site (Figure 1iv). An alternative interpretation of this result is that perhaps all T cells in these experiments exhibit a small bias towards the infection site, but only a small fraction of the cells display detectable significant bias. To test this interpretation we performed additional simulations. Specifically, we simulated movement of 500 cells by varying the strength of attraction towards the infection site (*κ*_*a*_) for all cells and the number of cell movements (assuming that every movement was recorded with 2 min timesteps), and assuming that cells start their search at 150 *μ*m from the parasite. For each combination of parameters, we fit a vMF distribution to each of 500 trajectories, generating a distribution of concentrations *κ*_*a*_. One interesting observation from these simulations is that as the bias towards simulated infection sites increases, the mean of the estimated concentration parameters increases as well, leading to a large difference between experimentally observed and simulated distributions (Figures 5A,B, Kolmogorov-Smirnov test *p* < 0.001). The simulated distributions typically had smaller tails than the experimental distribution, implying less variation in the estimated concentrations *κ*_*a*_ (Figures 5A,B), especially as *κ*_*a*_ increases past 0.1 (Figure 5C). This may partly be due to the simulated cells not having persistence in their movement, while real cells do appear to move with persistence that may lead a cell to move randomly in a continuous direction toward or away from the parasite. The differing sizes of the tails suggest that our data are not consistent with the idea that all cells exhibit some level of bias towards the infection site. Furthermore, power analyses suggest that to see at least 20*%* of cells exhibit statistically significant bias towards the infection, all cells must have a relatively substantial attraction (at least *κ*_*a*_ ≈ 0.5, Figure 5D). That consistently high attraction would result in a high average attraction not observed in actual data. Thus, our results are more consistent with the explanation that only a small fraction of T cells exhibit bias towards the parasite when there is already a cluster of T cells.

### 3.6 T Cells Only Display Exclusive Attraction to the Infection Site When a T Cell Cluster has Already Formed Around the Parasite

In our analysis, we focused on detecting if T cells are attracted to the site of infection, and our tests did not compare *Plasmodium*-specific cells with control T cells (specific to an irrelevant antigen) because those results could be biased if both malaria-specific T cells and T cells with irrelevant specificity may be equally attracted to the infection site. However, our vMF distribution-based metric could be too sensitive, detecting attraction when it does not exist. Indeed, we showed that for T cells moving with a correlated random walk, some cells may be detected as attracted to the infection in the absence of actual attraction (Supplementary Figure S6). We reasoned that if T cells are truly attracted to the infection site, them fewer cells (if any) should display attraction to areas distant from the actual parasite’s position.

**FIGURE 6:**
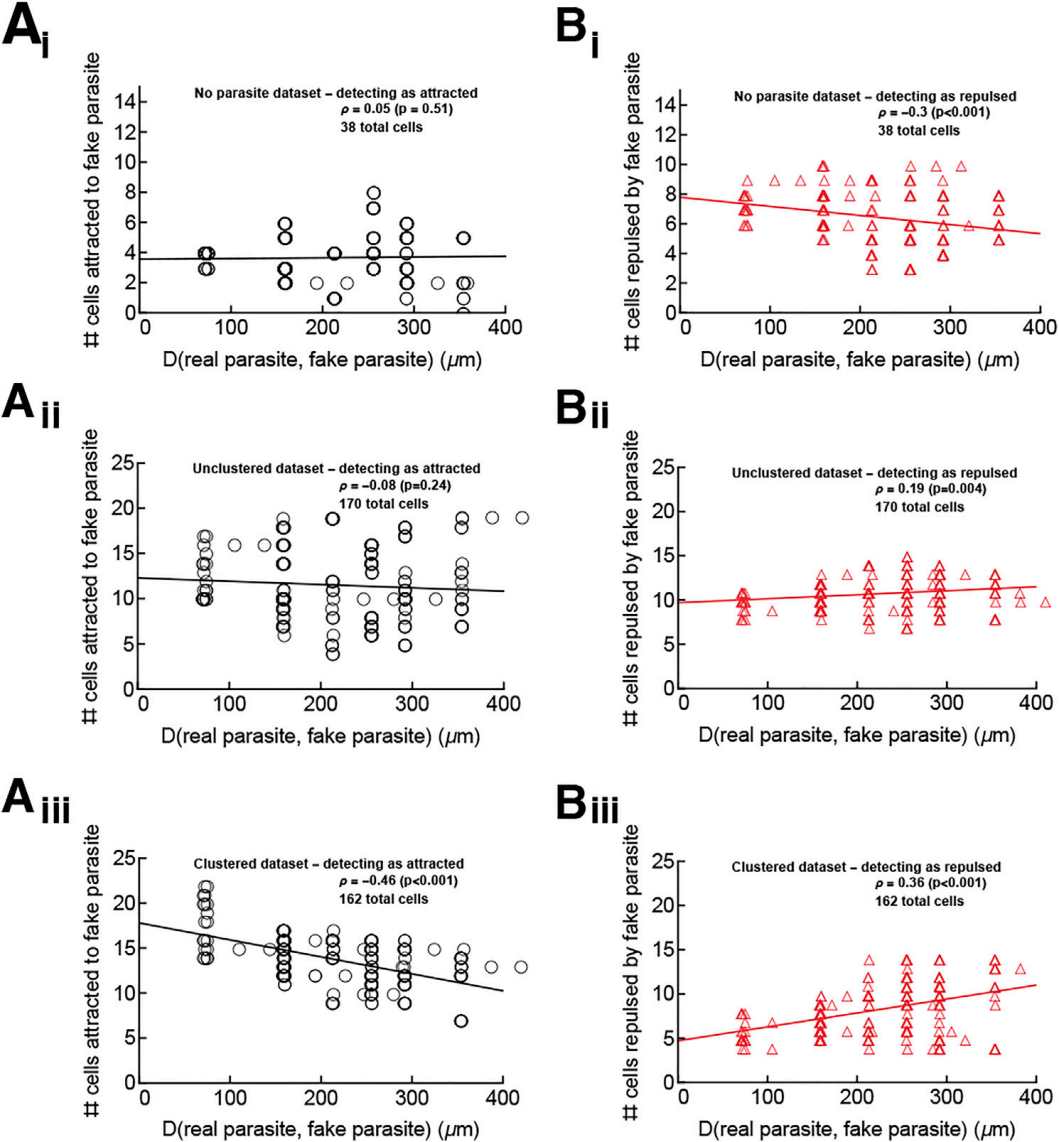
The parasite’s position is an attraction point for T cells only in the large clustered data. We performed analyses to test if cells in the “no parasite” data (panels i), in the unclustered/small clustered datasets (panels ii), and in the large clustered datasets (panels iii) display attraction to sites other than the infection site. We chose positions of a “fake” parasite every 10 *μ*m on the grid extending 250 *μ*m away from the real parasite. For the “no parasite” movie, which has no infection, the “true” parasite was assumed to be in the center of the imaging volume for the purposes of judging the distance of fake parasites from the true parasite. Then for every T cell we calculated the concentration parameter *κ*_*a*_ (using Eq. 1) and determined if the estimated parameter is different from 0 using LRT (see Materials and Methods for more detail). In this way, we calculated the number of T cells attracted to (*κ*_*a*_ > 0, panels **(A)** or repulsed from (*κ*_*a*_ < 0, panels **(B)**) the “fake” parasite and the distance *D* between each “fake” parasite position and the true parasite position (denoted on *x* axes in the panels). The changes in the numbers of cells detected as attracted or repulsed with distance *D* were tested using the Spearman rank correlation test (the rank correlation *ρ* and *p* values from the tests are indicated on individual panels). The lines from linear regression are shown for visual purposes only.

For this analysis we chose “fake parasite” positions equally spaced in the 500 × 500 × 50 *μ*m^3^ imaging box (a total of 216 positions) and determined the number of real T cells that were detected as attracted to (or repulsed from) each fake parasite location (tested using our vMF distribution-based metric). For the no parasite (uninfected mouse) and unclustered/small clustered (infected mice) datasets, the number of cells detected as attracted to (or repulsed from) any position was similar (Figures 6i–ii). We surmise that in these datasets, any attraction or repulsion detected to the real infection site is simply an artifact (Figures 6i–ii). This corroborates the conclusion that in the absence of large cluster formation, T cells were searching for the parasite randomly (or with a weak bias that was not detectable). In contrast, in the large clustered dataset, more cells are detected as attracted and fewer as repulsed for fake parasite positions around the real parasite (for which the distance between the real parasite and “fake” parasite is small), suggesting that there truly is some T cell attraction to the real parasite position when there are other T cells already present near the parasite (Figure 6iii).

### 3.7 A Lower Number of T Cells is Required for Sterilizing Protection if There Is Biased T Cell Movement Towards the Infection Site

**FIGURE 7:**
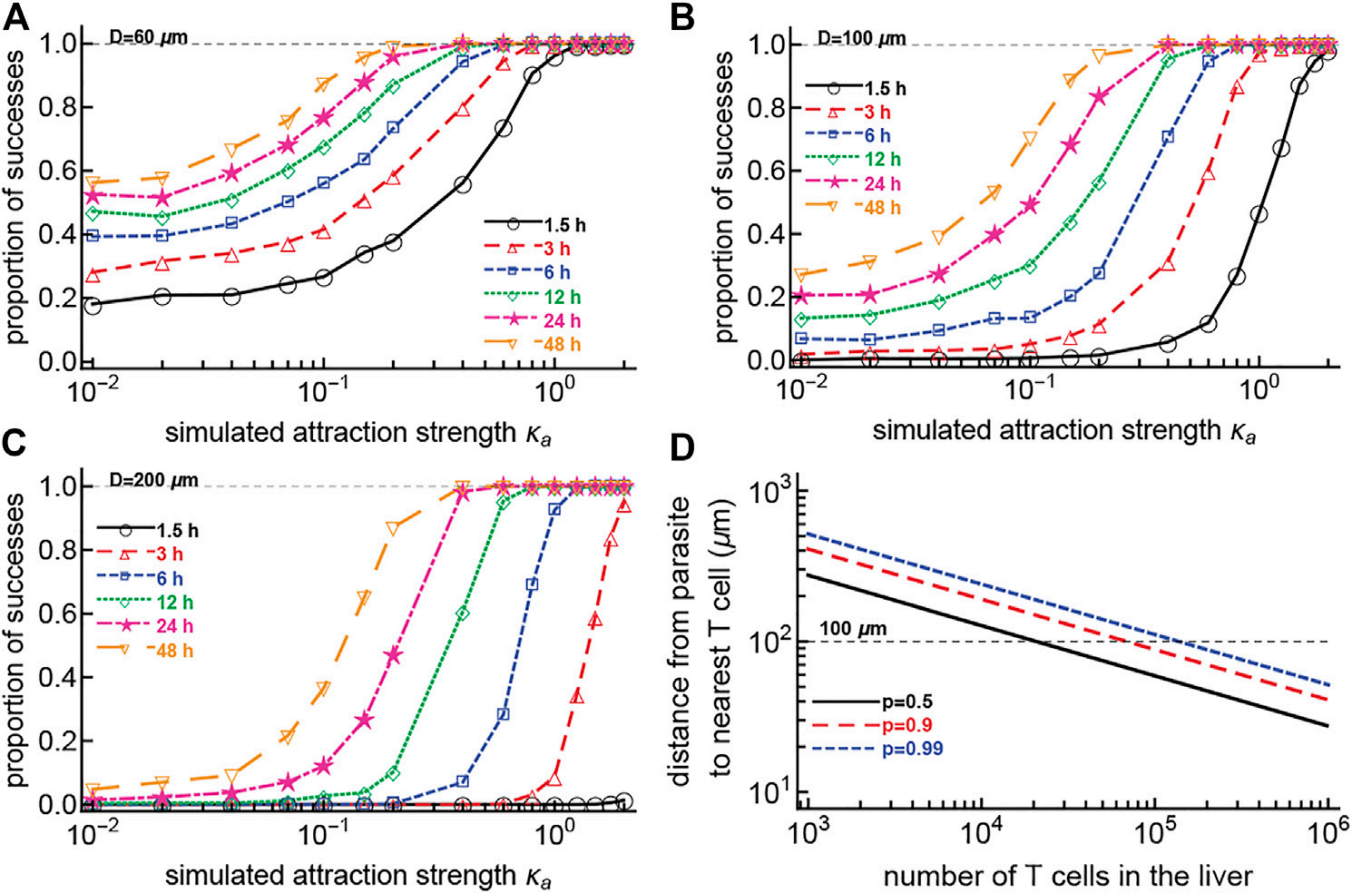
Small attraction dramatically increases the chances for a T cell to find the parasite within 48 h. We simulated CD8 T cells searching for a malaria liver stage assuming variable levels of T cell attraction to the parasite, defined by the concentration parameter *κ*_*a*_ of the vMF distribution. Cells started their search for the infection 60 **(A)**, 100 **(B)**, or 200 **(C)** *μ*m from the parasite. We calculated the probability that the cells reach the parasite within 40 *μ*m distance at various times after infection. To simulate a T cell walk we assumed that T cell movement lengths follow a generalized Pareto distribution (Supplementary Equation S8) with pseudorandomly chosen turning angles. We did simulations for 1,000 cells for each value of *κ*_*a*_. In panel **(D)** we calculated the distance between a parasite and the nearest CD8 T cell assuming T cells are randomly distributed in the 1 ml = 1 cm^3^ volume of the liver, and showed different levels of certainty that the cell is within that distance (defined by the probability *p*). The distance from the parasite to the nearest T cell is calculated as $d=\frac{r}{2}\sqrt[{3}]{\frac{\text{ln}\left(1/\left(1-p\right)\right)}{n}}$ , where $r=\sqrt[{3}]{3/\left(4\pi \right)}\text{ cm}$ is the radius of a sphere with volume 1 cm^3^ (this approximates a mouse liver) and where *n* is the number of T cells in the liver. For *p* = 0.99 when there are *n* = 10^5^ T cells in the liver, the chance that 1 T cell is at the distance of *d* = 100 *μ*m from the parasite is 99%.

If CD8 T cells search for the malaria liver stages nearly randomly, we wondered how the presence of weak attraction (that is not detectable in current experiments) could change the ability of T cells to find the parasite (i.e., reach within 40 *μ*m of the parasite). We performed 1,000 simulations for each combination of varying T cell attraction to the parasite (the concentration *κ*_*a*_) and starting distance from the cell to the parasite, and counted the cells which reach the parasite (within 40 *μ*m) within a defined time period. Our results suggest that a small change in attraction strength *κ*_*a*_ can dramatically increase the chance of T cells finding the parasite, especially when the initial distance between the T cell and parasite is large (Figures 7A–C). Importantly, a relatively weak bias towards the infection (at least *κ*_*a*_ ≈ 0.2–0.3) is sufficient for T cells to locate the parasite within 48 h after infection, even for a relatively large initial distance between the T cell and the parasite (Figure 7C).

In our experiments, the initial distances between T cells and the parasite varied dramatically; the average was around 150–200 *μ*m (e.g., Figure 2). Given a mouse liver volume of about 1 ml (Marino, 2012), we calculated that if there are 10^5^ randomly distributed T cells in the liver [a number found experimentally to provide sterilizing protection against malaria in mice (Gola et al., 2018; Olsen et al., 2018)], the distance between a randomly positioned parasite and its nearest T cell is about 100 *μ*m, with 99% probability (Figure 7D). However, without biased movement towards the parasite, CD8 T cells located at 100 *μ*m from the parasite are unlikely to locate the infection even within 48 h (Figure 7B), suggesting that some weak attraction may be guiding T cell search for the infection.

Our simulations so far focused on scenarios where a single T cell reaches the parasite. However, when multiple T cells search for the parasite, the probability of at least 1 T cell finding the infection could be higher. To investigate this, we performed 1,000 simulations to allow several T cells to search for the parasite by varying the strength of T cell attraction to the parasite (concentration *κ*_*a*_), the number of searching T cells, and the length of search, and by fixing the starting distance of T cells from the parasite to 150 *μ*m. For each combination of parameters, we determined the proportion of the 1,000 simulations for which at least 1 T cell reaches the parasite (within 40 *μ*m). Results suggest that for essentially all parasites to be found in 48 h, we need around 10 T cells per parasite and a weak attraction defined by a concentration of at least *κ*_*a*_ ≈ 0.2 (Supplementary Figure S16). These findings suggest that when multiple T cells search for an infection, weak attraction greatly improves the chances of T cells finding the parasite.

## 4 Discussion

It has been well established that activated and memory CD8 T cells are capable of providing sterilizing protection against infection of *Plasmodium* sporozoites in mice (Schmidt et al., 2008; Schmidt and Harty, 2011; Fernandez-Ruiz et al., 2016). However, the ways that T cells locate and eliminate all *Plasmodium*-infected hepatocytes remain unclear. By generating unique data on the movement of liver-localized activated CD8 T cells within a few hours after infection with *Plasmodium* sporozoites, and by rigorous analyses of these data using a newly developed metric based on the von Mises-Fisher distribution, we found that most CD8 T cells search for the infection site randomly. Using stochastic simulations, we showed that randomly searching T cells have a high failure rate of finding parasites. A small bias towards the infection dramatically improves the chance of T cells finding the infection, and thus reduces the number of liver-localized T cells needed for sterilizing protection. Power analyses showed, however, that current experimental set-ups allowing for intravital imaging of livers of live mice for only a few hours do not generate sufficient data to detect attraction to the infection site by individual T cells.

For all analyzed datasets, we consistently found that a small fraction (about 15–20%) of T cells displayed strong movement bias to the infection site; such biased T cells included both T cells attracted to the parasite and T cells repulsed from the parasite. In the case where there were several T cells near the parasite (large clustered data), the number of T cells attracted to the infection site was significantly higher than that of repulsed cells. The distance between the T cell and the infection did not correlate with the strength of attraction; however, the speed at which T cells moved was a strong predictor of attraction, that is, more rapidly moving T cells displayed stronger bias. This observation highlights the need for a better understanding of the mechanisms that regulate the movement speed at which T cells survey peripheral tissues. In our case, however, we found that rapidly moving T cells having a higher bias towards the infection site may be an artifact of a correlated/persistent random walk.

The reasons why some T cells (e.g., OT1 cells in the large clustered data) display attraction towards the infection site while others (e.g., P14 cells in the large clustered data) search randomly are unclear. It is generally believed that movement patterns of T cells in tissues are regulated by chemokines, i.e., T cells with appropriate chemokine receptors follow chemokine gradients in the environment (Krummel et al., 2016b). However, it is not yet understood if T cells indeed follow chemokine gradients (which may be shallow in many circumstances) or if chemokines simply regulate the cells’ velocities (Okada et al., 2005b; Ariotti et al., 2015; Krummel et al., 2016b). For example, CXCL21 chemokine and CCR7 receptors on B cells regulate the localization of B cells near B-T zones of lymph nodes (Okada et al., 2005b). However, while the CXCR3 receptor is important for CD8 T cell movement in the skin or brain (Harris et al., 2012; Ariotti et al., 2015), whether T cells in these tissues follow the chemokines’ gradients has not been established. We recently found that the LFA-1 receptor on T cells is critical for T cell motility in the liver; however, even LFA-1-deficient T cells are capable of locating *Plasmodium* liver stages with a somewhat lower success rate (McNamara et al., 2017). Whether receptors regulate T cell attraction to the *Plasmodium* liver stages is unclear. It is possible, however, that such attraction is achieved by a combination of receptors, and the lack of any single receptor does not dramatically impact the ability of T cells to locate the infection. It was recently found that the migration of *Mycobacterium tuberculosis*-specific CD4 T cells from the lung vasculature into parenchyma is dependent on many receptors, and lacking a single receptor had only a moderate to minimal impact on the rate at which T cells migrate from the blood to the lung (Hoft et al., 2019).

The presented results have several limitations. Even though our intravital imaging experiments lasted for 2–3 h, the amount of data collected was not sufficient to detect the weak attraction that individual T cells may exhibit when searching for the parasite. Experiments with a longer duration may be ethically difficult, and long-term imaging of surgically exposed livers may also generate artifacts due to tissue damage. The imaging frequency in our data was relatively low (1.5–2 min per stack of images) in order to allow for longer movies with lower laser exposure to the liver. While more frequent measurements could increase the number of data points, long movies may increase chances of tissue damage, require time-consuming processing steps, and result in a bias of imaged cells remaining in the imaging volume for a long time. Therefore we aimed for a balance between the length of imaging and the costs associated with longer movies.

Due to the limitations of intravital imaging, we could only record cell positions in a limited 3D volume of approximate 500 × 500 × 50 *μ*m^3^ centered near a parasite. If a cell exits that volume we no longer record its positions, so there may be bias toward recording more movements from T cells that act attracted to the parasite than cells that act repulsed from the parasite, since repulsed cells are more likely to leave the imaging volume. We performed simulations that approximately allow us to correct for such bias in our vMF distribution-based metric.

While it is not often mentioned, intravital imaging may induce local damage due to exposing tissues to lasers, especially in longer experiments. It is often difficult to evaluate the degree of liver damage any specific experiment involves. In our experiments we observed that in *Plasmodium* sporozoite-infected livers, CD8 T cells move relatively slowly, with speeds of 1.5–2.5 *μ*m/min. This was significantly slower than the 3.5 *μ*m/min estimated in one control movie without an infection (“no parasite” dataset), and lower than the average T cell speeds estimated in previous studies, which are in the range of 5–10 *μ*m/min (Guidotti et al., 2015; McNamara et al., 2017). One potential explanation of this difference may be due to the frequency of imaging, which in our experiments was rather low; evidence that imaging frequency has a direct influence on the inferred speeds of cells will be presented in a future work.

For some of our analyses, we pooled together the movements of different cells found in different mice to increase the analyses’ power. In general we found similar results (although at times statistically not significant) as when we analyzed the individual datasets separately. The vMF distribution, while a straightforward representation of bias in 3D movement, may not perfectly approximate the distribution of angles towards the infection, although it is close (Supplementary Figure S12). We found that in cases when the vMF distribution does not fit the angle to infection data well, a mixture of vMF distributions can match the data very well (Supplementary Figure S12A–C). Such an approach also allows us to estimate the fraction of angles near 90° that deviate from the single vMF distribution; what such angles represent biologically has yet to be determined. For example, this pattern may arise if cells attempt to approach a particular location and are unable to do so directly due to the absence of a direct path to the location; in this case the cells may thus meander.

Most of our simulations were concerned with the process of T cells finding the parasite; however, to protect from malaria, T cells must also kill the parasite. The time it takes for CD8 T cells to kill *Plasmodium* liver stages after reaching them has not been quantified, and therefore our estimates of the search time should be considered minimum estimates of the time it takes for T cells to find and kill the parasite.

One major limitation is that we did not take into account the fact that liver-localized CD8 T cells move in liver sinusoids (McNamara et al., 2017; Rajakaruna et al., 2020) and are thus constrained in their movement. The chance of randomly finding an infection may be influenced by the structure of the sinusoids (Rajakaruna et al., 2020), and it remains to be determined how a biased search will find parasites in a constrained sinusoid structure instead of the open (3D) space in which all the metrics in this paper operate. An argument could be made that ignoring liver sinusoids makes the simulations not useful for approximating cell movement; however, as we choose simulation parameters to replicate real movement, we think our simulations, even though performed in open space, are an acceptable representation of cell movement in the liver. How CD8 T cells may search for the infection site in the constrained liver environment of sinusoids will be presented in a future work.

One of the purposes of this work was to test predictions of our recently published density-dependent recruitment model of CD8 T cell cluster formation around *Plasmodium* liver stages, which states that the formation of clusters is driven by a positive feedback loop where larger clusters recruit more T cells (Cockburn et al., 2013; Kelemen et al., 2019). While the data are supportive of a random search of T cells for the parasite, it is still unclear why some parasites already have T cells nearby at the start of imaging while other parasites have no T cells nearby or there is no accumulation of T cells near other parasites in ∼1–2 h of imaging. Even in situations in which the first T cell discovers the parasite, we found no evidence that T cells in surrounding areas become more attracted to the parasite within an hour. The data thus do not yet allow us to discriminate between models with density-dependent recruitment and with variability in attraction to parasites (Kelemen et al., 2019).

There are several ways the work outlined in this paper can be extended. We did not investigate if any specific chemokine receptors (e.g., CXCR3, CCR5, or CX3CXR1) impact the success rate of the T cell search for the infection site. This is one focus of our current research. Including the structure of liver sinusoids in simulations of T cell searches for infection would likely provide more realistic estimates of the time it takes for T cells to find the parasite. To measure T cell attraction to the infection in this paper, we used the vector from the T cell to the parasite as the optimal route to reach the infection site. However, due to physical constraints from the liver sinusoids, the optimal route to the infection may not be the direct vector. By imaging the parasite, T cells, and liver sinusoids, it may be possible to quantify an optimal route, traveling through the sinusoidal structure, and to determine if the cells move with attraction along the structure. Work on this project is underway.

Future work may need to investigate how quickly T cells form clusters around the parasites and if the degree of attraction we estimate in this work is sufficient to explain the formation of relatively large clusters (e.g., 5–10 CD8 T cells) near individual parasites (Cockburn et al., 2013; Kelemen et al., 2019). Similarly to how we used statistics to predict the distribution of angles to the parasite using the vMF distribution, we also attempted to find an analytical description of a distribution of changes of distances to the parasite with T cell movement. However, that distribution is more complicated mathematically, and we left it for future work. Our analysis propounds that the speed at which T cells search for an infection may be correlated with the ability of T cells to locate the infection site. However, there appears to be a trade-off between the speed T cells are moving in tissues and the ability of such T cells to process environmental signals to detect the infection—too rapid cells may miss many of the infected cells in their haste (Mrass et al., 2017). Whether there is an optimal movement pattern of T cells to locate all infections in a small enough time to cure infection is also work for a future project (Moses et al., 2019). Despite potential limitations, our work provides novel data from innovative imaging experiments and rigorous mathematical analyses that begin to elucidate how CD8 T cells search for infection in the complex tissue of the liver.

## Data Availability

The original contributions presented in the study are publicly available. This data can be found here: https://github.com/viktorZenkov/measuringAttraction/blob/master/Data/AllPositionData.xlsx and, in csv format, https://github.com/viktorZenkov/measuringAttraction/blob/master/Data/AllPositionData.csv. These files contains T cell movement/track data both generated for this paper and published previously. Also, we provide two Imaris files that contain original imaging data and objects (Spots) for unclustered/small clustered and large clustered datasets (https://doi.org/10.5281/zenodo.5715658). Coding information and access can be found in the article.

## Abbreviations

vMF, von Mises-Fisher; GFP, green fluorescent protein; TCR, T cell receptor; OVA, ovalbumin; LCMV, lympocytic choriomeningitis virus; LRT, likelihood ratio test; OU, Ornstein-Uhlenbeck.
