# Supplementary material for: A New Method Based on the von Mises-Fisher Distribution Shows that a Minority of Liver-Localized CD8 T Cells Display Hard-To-Detect Attraction to Plasmodium-Infected Hepatocytes
Source: Front Bioinform. 2022 Jan 31;1:770448. doi: 10.3389/fbinf.2021.770448 (PMC9580869; doi:10.3389/fbinf.2021.770448)
Supplement: Supplementary file 1 [file DataSheet1.pdf]

A new method based on the von Mises-Fisher distribution shows that a minority of liver-localized CD8 T cells display hard-to-detect attraction to *Plasmodium*-infected hepatocytes

Viktor Zenkov, James O'Connor, Ian A. Cockburn, and Vitaly V. Ganusov

Supplemental Information

Additional experimental details

Mice were prepared for two-photon microscopy as described in previous work [1]. In summary, mice were anesthetized with a mix of Ketamine (100 mg/kg) and Xylazine (10 mg/kg). Throughout the surgery and imaging pro cedure the mouse temperature was maintained at 37°C using a heating mat attached to a feedback probe inserted in the mouse rectum. A lateral incision was made over the left lobe of the liver and any exposed vessels cauterized by applying light pressure to the vessel until clotting occurred naturally. The mouse was then placed in a custom made holder. The liver was then exposed and directly adhered to a cover slip that was secured in the holder. Once stable the preparation was transferred to a Fluoview FVMPE-RS multiphoton microscope system (Olympus) equipped with a XLPLN25XWMP2 objective (25x; NA1.05; water immersion; 2mm working distance). The laser was 860 nm with a tissue penetration of approximately 50 μm. Coloring depended on the content of the experiment: for experiments with 2 colors, we used violet and green (BA495-540nm + BA410-455nm), while for experiments with 3 colors, we used red, violet, and green (BA575-645nm + BA495-540nm + BA410-455nm). For analysis of the motility of the sporozoites and liver-localized CD8 T cells, we had a tissue penetration depth of approximately 50 μm in a z-stack (2 μm/slice), typically acquired using a standard galvano-scanner at a rate between 0.5 to 1 stacks of 23 images/slices per minute depending on the movie.

Imaging volumes were chosen based on the presence of a parasite; since the Olympus software generates little background autofluorescence and the parasites do not move, this process was dependable. The choice of which parasites to image was contingent upon their appearing in clear, healthy sections of liver tissue, and the proximity of T cells at the time of imaging. Each parasite chosen for imaging required unimpeded blood flow in surrounding sinusoids, bright autofluorescence, and clear surrounding structures. In addition, the parasites chosen for imaging depended on what information we wanted. For the 0 hour timepoint, parasites were selected if they had healthy tissue and had no T cells within a 40 μm radius of the parasite. This allowed for an analysis of attraction of T cells with no primary signaling from an infected cell. At 2 hours post infection, parasites were selected if they had healthy tissue and had ≥ 1 T cells within a 40 μm radius of the parasite. This allowed for an analysis of attraction of T cells with at least 1 primary cell signaling from an infected hepatocyte. We used the 40 μm radius because it has been used to represent the average width of a standard murine hepatocyte [2].

Imaging data processing details

We analyzed raw imaging data using the Imaris x64 software (Bitplane) v9.2. We performed the tracking of individual cells in a z-stack using the "Spots" function in surpass mode in Imaris. Extensive manual adjustments were subsequently done to ensure the accuracy of the tracks. The detection

of individual cells relied upon their relative fluorescence intensity and size (diameter ≥ 9 μm), with a max gap size of 3. We used an autoregressive motion algorithm with background subtraction, tracking enabled, fill gap enabled, and no region growing in Imaris. Since cells are not all in the imaging volume at all times, we used a variable track duration. The detection of fluorescence around each spot was manually adjusted in Imaris prior to tracking cells to reduce background detection at each time point, ensuring the clear distinction of cell versus autofluorescence by the algorithm. Two Imaris files are provided at `https://doi.org/10.5281/zenodo.5715658`, with one file for the unclustered/small clustered dataset and one file for the large clustered dataset.

Datasets

1. Dataset #1: unclustered/small clustered data (OT1 T cells in Pb-CS^{5M}-infected B6 mice). No or few T cells were near the parasite.
2. Dataset #2: large clustered data (OT1 T cells in Pb-CS^{5M}-infected B6 mice). Several T cells were found near the parasite (i.e., with T cell cluster).
3. Dataset #3: no parasite data (OT1 T cells in naive/uninfected B6 mice).
4. Dataset #4: Paris data (PyTCR cells in Py-infected Balb/c mice).
5. Dataset #5: co-clustered ("Kelemen") data (PyTCR and OT1 cells in Py-infected CB6 mice).

Metrics

We define four metrics to measure T cell attraction towards the parasite (Figure S3).

1. Metric 1: the angle metric.
   (a) For a movement of a cell between timepoints, the angle of movement is defined as the angle between two vectors: the vector from the cell's position before it moves to the parasite and the vector from the cell's position before it moves to the cell's position after it moves.
   (b) An acute angle corresponds to "getting closer" and an obtuse angle corresponds to "getting farther". The probability of randomly getting closer is 0.5.
   (c) To test, we associate a Bernoulli distribution with probability 0.5 with each movement, then sum up the distributions for all the movements to get a binomial distribution. Our null distribution is then a binomial distribution with $n =$ the number of movements and $p = 0.5$.
2. Metric 2: the improved change of distance metric.
   (a) For a movement of a cell between timepoints, the change of distance is defined as the distance from the cell to the parasite after the cell moves minus the distance from the cell to the parasite before the cell moves.
   (b) A negative change of distance corresponds to "getting closer" and a positive change of distance corresponds to "getting farther". The probability of randomly getting closer is $1/2 - r/4x$ (Figure S4).

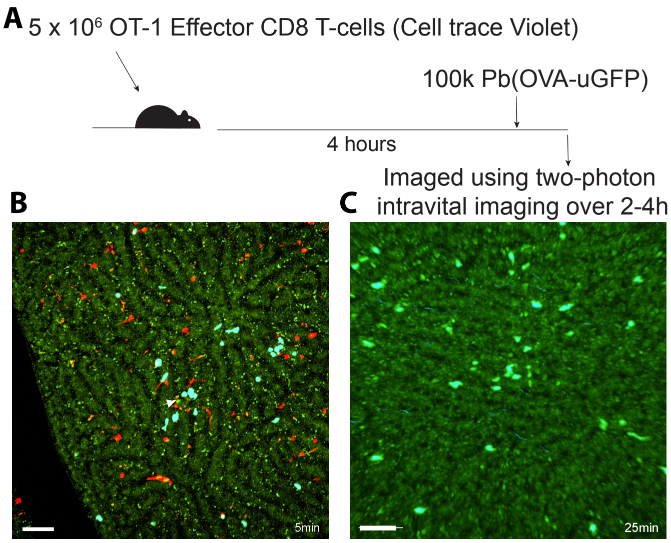

Figure S1: We investigate T cell dynamics in the liver following infection with *Plasmodium* sporozoites. Four hours after i.v. cell transfer of 5×10^6 cell trace Violet labelled OT1 CD8 T cells (blue), mice were injected with 1×10^5 *Plasmodium berghei* Pb(OVA-uGFP) and immediately prepared for intravital imaging (panel A). We imaged the mice using a two-photon standard galvanometer scanner in order to acquire a 50 μm deep Z-stack. We show representative images of small clustering (panel B) and large clustering (panel C) image acquisitions. Representative videos of small clustering (movieS1) and large clustering (movieS2) are in the supplemental materials, as well as the Imaris files at `https://doi.org/10.5281/zenodo.5715658`. The green sporozoite is highlighted with a white arrow in both images. The scale bars are 20 μm. The numbers overlaid at the bottom right of each image are the timestamps in minutes.

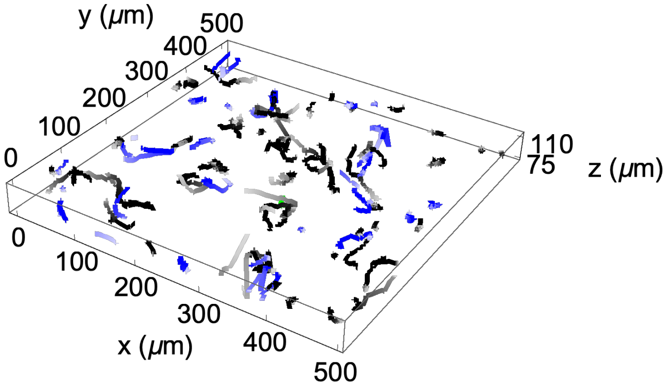

Figure S2: Display of CD8 T cell movements in the liver over time from one unclustered/small clustered dataset. Blue tracks are *Plasmodium*-specific T cells (OT1) and black are nonspecific T cells (P14), with the parasite shown in green (with coordinates (290, 210, 76)). Tracks are shown over time, with early timepoints lighter in color than later timepoints. The data can be explored in greater detail in an interactive Unity environment at `https://viktorzenkov.github.io/measuringAttraction`.

(c) To test, we associate a Bernoulli distribution with $p = 1/2 - r/4x$ with each movement, then sum up the distributions for all the movements to get a Poisson binomial distribution, and this is our null distribution.

3. Metric 3: the angle distribution metric.

   (a) The angle distribution metric uses the angle of movement from Metric 1 and the angle version of the von Mises-Fisher distribution (described later).

   (b) Taking a dataset of angles, we use a log-likelihood test to determine the concentration parameter for the angle version of the von Mises-Fisher distribution most likely to generate the directions corresponding to the angles.

   (c) If this distribution's test statistic is significantly different from a random test statistic, then that corresponds to biased movement, and if it is not significantly different then that corresponds to unbiased movement. If a cell is biased with $\kappa_a > 0$, then the cell is considered attracted, and if a cell is biased with $\kappa_a < 0$, then the cell is considered repulsed.

   (d) A followup test uses the multinomial distribution. For a set of cells, each of which has had its bias ascertained as attracted, repulsed, or unbiased, we set our null distribution to a multinomial distribution with probabilities set to the proportion of cells detected as unbiased, p_u, and the other probabilities each set to $\frac{1-p_u}{2}$. This takes into account the proportion of cells that are unbiased while still allowing a test between attracted and repulsed quantities.

4. Metric 4: average angle metric.

   (a) The average angle metric uses the angle of movement from Metric 1.

   (b) To test, we use a Student T test to compare the mean of the angles for a cell to 90 degrees, which represents randomness.

Statistical/computational analyses

A randomly moving cell is more likely to have its distance from a parasite increase. There have been few studies that rigorously address the question of how to detect attraction of a moving lymphocyte towards an infection site. Commonly, two metrics or their variations have been used: the angle-based metric and the distance-based metric [3–5]. According to these metrics, cells moving towards the parasite exhibit acute angles to the parasite and reduction in distance to the parasite, respectively (Figure S3A&B). The natural assumption is that randomly moving (unbiased) cells should exhibit a similar number of movements towards and away from infection. However, as far as we know this assumption has not been thoroughly explored.

To evaluate the potential bias of T cells towards the infection site, we performed stochastic simulations in which T cells searched for the infection. Simulations showed that while the angle metric is not biased (i.e., for randomly moving T cells, there are similar proportions of acute and obtuse angles to infection), the distance metric is biased (i.e., there are more movements away from the infection than towards the infection). (Note that this usage of the word "bias" refers to a skewed result, unlike our other usage, which refers to cell movement being attracted or repulsed.) Even before

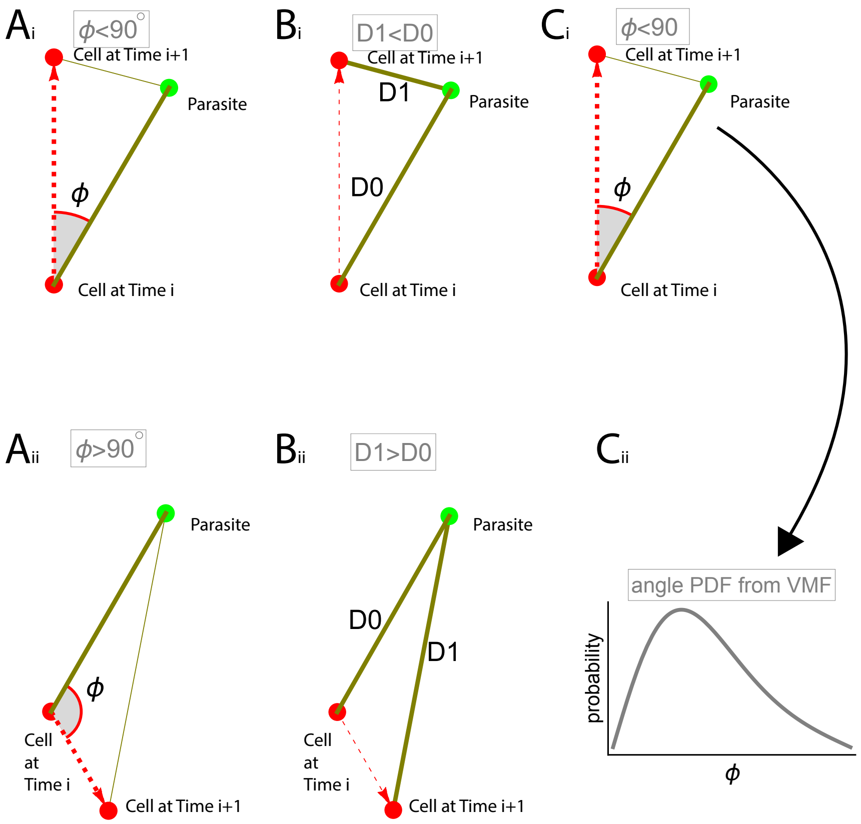

Figure S3: Four metrics were used to test for attraction of CD8 T cells towards the infection site. Panels A: the angle metric is defined as the angle between the vector from the cell to the parasite and the vector formed by the cell's movement. An acute angle corresponds to "getting closer" (Ai) and obtuse corresponds to "getting farther" (Aii). The fourth metric, the "average angle" metric, uses these same angles. Panels B: the distance metric is defined as the change between the distances from the cell to the parasite before and after the cell moves. A negative change of distance corresponds to "getting closer" (Bi) and positive corresponds to "getting farther" (Bii). Panels C: the angle distribution metric takes into account the actual values of angles and compares these values with the von Mises-Fisher distribution (eqn. (1)) leading to an estimate of the concentration parameter κ_a (a measure of attraction). If $\kappa_a > 0$ and this distribution's test statistic is significantly different from a random test statistic ($p < 0.01$), we consider the cell to have "attraction". If the test statistic is not significantly different, then we consider the cell to not have bias. If $\kappa_a < 0$ and the test statistic is significantly different, then we consider the cell to be repulsed.

performing simulations, graphing movement possibilities of T cells with respect to the infection site revealed that for a finite distance between the cell and the infection, there are more chances for the T cell to move away than to get closer (Figure S4). Defining r as the distance that the cell moves and x as the distance from the cell to the parasite, the probability that the distance decreases for a T cell movement is $\frac{1}{2} - \frac{r}{4x}$. The subset of the surface of the r-sphere located inside the x-sphere are the positions for which the cell's distance to the parasite gets smaller, and the subset of the surface of the r-sphere located outside the x-sphere are the positions for which the cell's distance to the parasite gets larger. Note that if $r > 2x$, causing the fraction to be less than 0, then the cell moves so far that none of its potential destinations are closer to the parasite than its current location, which means the probability that the distance gets smaller is 0 (Figure S4).

Thus, the distance metric is biased in the sense that movements have a less than 50% probability of getting closer to the parasite, which means our null distribution cannot define randomness as

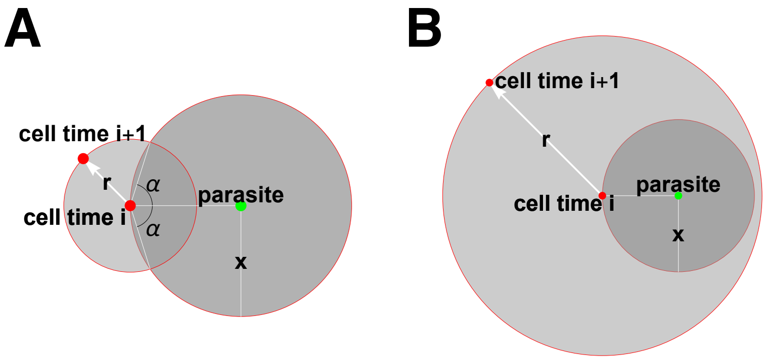

Figure S4: This graphic demonstrates the bias in the distance metric's measure of attraction to the infection site. Based on the volumes of the areas the cell may move to, the probability that a T cell moves closer to the parasite is lower than the probability that the T cell moves away from the parasite. With r defining the distance that the T cell moves and x defining the distance between the cell and the parasite, the probability that the distance between a randomly moving T cell and the parasite declines with the movement is $P(\Delta D < 0) = 1/2 - r/(4x)$ for $r < x$ (Panel A) and is $P(\Delta D < 0) = 0$ if $r > x$ (Panel B).

50%. To form a corrected test, we utilized a Poisson binomial distribution as our null distribution, which is a sum of Bernoulli distributions with different probabilities (in our case, a Bernoulli for each movement). The probability that k movements (out of n) are towards the parasite is then:

$$P(k) = \sum_{A \in F_k} \prod_{i \in A} p_i \prod_{j \in A^c} (1 - p_j), \tag{S1}$$

where F_k is the set of all subsets of k integers that can be selected from $1, 2 \ldots n$ and A^c is the complement of A, $p_i = 1/2 - r_i/(4x_i)$ and r_i is the length of the ith cell movement and x_i is the distance between the T cell and the parasite before the ith movement (Figure S4). With this correction, the angle-based and distance-based test results are numerically indistinguishable.

The von Mises-Fisher distribution provides a way to model and measure attraction. One of the limitations of the angle- and distance-based metrics is that the actual value of the angle/distance is ignored and converted into a boolean value, thus potentially reducing the power of detecting bias in cell movement. To retain the actual values of the angles in a test, we used the von Mises-Fisher (vMF) distribution. The vMF distribution is a natural extension of the von Mises distribution used in ecology, which is restricted to describing bias in 2D [6].

The vMF distribution chooses an n-dimensional (we use $n = 3$) vector given a direction vector μ and a concentration parameter κ [7, 8]. Sampling from the vMF distribution provides a vector chosen pseudorandomly with a bias toward the given vector whose strength is dependent on κ, with $\kappa \to 0$ having no bias, $\kappa > 0$ having positive bias (attraction), and $\kappa < 0$ having negative bias (repulsion). The probability density function for a vector χ in the vMF distribution in 3D is

$$P(\chi | \mu, \kappa) = \frac{\kappa e^{\kappa \mu^T \chi}}{2\pi (e^{\kappa} - e^{-\kappa})}, \tag{S2}$$

where μ is the direction vector toward which there is bias and $|\mu| = |\chi| = 1$. To calculate the angle

ϕ between vectors χ and μ, we let $\mu = (0, 0, 1)$ and $\chi = (x, y, z)$. Given that $|\chi| = 1$, the angle ϕ then only depends on the value of z and can be calculated from the relationship $\cos(\phi) = z$. With that transformation and utilizing Mathematica we found the following probability density function for the angle ϕ:

$$P(\phi|\kappa) = \frac{\kappa \sin(\phi) \mathrm{e}^{\kappa \cos(\phi)}}{2 \sinh(\kappa)}, \qquad 0 \leq \phi \leq \pi, \tag{S3}$$

where ϕ is the angle between the vector to the attraction point and the cell movement vector. Note that in a case with no attraction, $\lim_{\kappa \to 0} P(\phi|\kappa) = \frac{1}{2} \sin(\phi)$. Because the magnitude of the concentration parameter is not intuitive, another useful parameter is the fraction of acute angles towards the attraction site. This fraction is found by integrating the vMF distribution

$$f_a(\kappa) = \int_0^{\pi/2} P(\phi) \mathrm{d}\phi = \frac{1}{1 + e^{-\kappa}}, \tag{S4}$$

where $f_a(0) = 1/2$ and $f_a(\infty) = 1$.

Estimating the concentration parameter of the vMF distribution. First, we sum the logs of the probabilities of getting each experimentally determined angle to the parasite ϕ_i from the vMF angle PDF (eqn. (1)) to get the negative log-likelihood function

$$\mathcal{L}(\kappa) = -\sum_{i=1}^{n} \log \Big(P(\phi_i|\kappa_a) \Big), \tag{S5}$$

where n is the number of movements in the data. The maximum likelihood estimate κ_a is found by minimizing eqn. (S5) (e.g., in Mathematica using the function `Maximize` on the inverse of the expression). Whether the found estimate $\hat{\kappa}_a$ is different from 0 is determined by using a likelihood ratio test (LRT) to compare $\mathcal{L}(\hat{\kappa}_a)$ and $\lim_{\kappa_a \to 0} \mathcal{L}(\kappa_a)$ [9].

It is straightforward to use a mixture of vMF distributions to explain angle distribution data. In particular, we found that while a single vMF distribution describes the distribution of angles to the parasite reasonably well, there is an over-representation of 90° angles in the data (e.g., Figure S12). Therefore, in this case we fit the following mixture distribution to the data

$$P(\phi|\kappa, f, n) = f \frac{\kappa \sin(\phi) \mathrm{e}^{\kappa \cos(\phi)}}{2 \sinh(\kappa)} + (1 - f) \sin^n(\phi), \tag{S6}$$

where f is the fraction of cells that are biased towards the infection, $\sin(\phi)$ is proportional to a vMF distribution with no bias, and n is used to explain the over-representation of angles at 90°. Improvements of this model fit over the simpler model, based on a single vMF distribution, are tested using a likelihood ratio test.

Modeling T cell attraction to the parasite. We use the vMF distribution to model T cell attraction to the parasite by using angles to infection as the set of angles, and we define κ_a as the

concentration parameter determining the strength of this attraction.

Modeling a correlated (persistent) random walk. Previous studies of cell movement employed various methods to simulate a persistent random walk, such as assuming a normal distribution of turning angles with mean 0 and some variance [10, 11]. Other studies used the Ornstein-Uhlenbeck model to simulate persistent random walks [12–14], which we also model in a later section. Alternatively, we use the vMF distribution to model persistent random walks using the concentration parameter κ_t for turning angles. In this model, turning angles are sampled from a vMF distribution with concentration κ_t. There is a direct relationship between the average turning angle $\bar{\phi}_t$ of a cell moving in a persistent random walk and the concentration κ_t:

$$\bar{\phi}_t = \int_0^\pi \phi P(\phi|\kappa_t)\mathrm{d}\phi = \frac{\pi \cosh(\kappa_t)\Big(I_0(\kappa_t) - \mathrm{e}^{-\kappa_t}\Big)}{2}, \tag{S7}$$

where $I_0(\kappa)$ is the Bessel function of the first kind of order 0.

The von Mises-Fisher distribution-based test may also be improved to eliminate bias occurring in a correlated random walk. The vMF distribution-based metric is not biased if cells move randomly with respect to a cell's previous movement. However, in many situations T cells move with a persistent random walk[15]: cells move in a direction positively correlated with the previous movement direction. To investigate if a persistent random walk may introduce bias in estimating attraction towards the infection site, we performed a set of simulations in which we describe cells' movement by our vMF distribution: the angle ϕ determines the cell's turning angle and the concentration parameter κ_t indicates the degree of the cell's persistence in the random walk. Analyses suggested that a persistent random walk can cause some cells to appear attracted to the infection site (or repulsed from the infection site) even though there was no actual attraction or repulsion (Figure S6).

Interestingly, we found that the fraction of cells detected as biased in simulations depended on the degree of walk persistence and the size of the volume in which the movement of T cells was considered, but in different manners. For low values of κ_t (low persistence), the fractions of cells detected as attracted and repulsed were similar (both low). For higher values of κ_t (high persistence), the fraction of cells detected as attracted was not impacted by the size of the volume, but the fraction of cells detected as repulsed was impacted: in all simulations with the maximum simulated persistence $\kappa_t = 2$, about 6% of cells were detected as attracted, while the percent of cells detected as repulsed ranged from about 6% to about 11% as the volume increased from a small box (Figure S6Aii) to an infinite volume (Figure S6Cii). This is not surprising, however, because cells that are detected as repulsed tend to move away from the parasite and are more likely to exit smaller imaging volumes (as in the smaller box in Figure S6Ai).

An interesting but perhaps unrealistic imaging experiment would be a situation when there is no "imaging" box. According to the simulations, this scenario results in the greatest disparity between the fractions of cells detected as attracted and repulsed. This phenomenon occurs for cells with greater persistence because of the artifact that cells moving away from the parasite tend to continue to move away, while cells moving towards the parasite tend to pass the parasite and then also move away. We could not derive an analytical expression to correct for the bias in detecting attraction of

cells moving in a correlated random walk. However, by simulating the movement of cells using the cell's initial position, movement length distribution, and turning angles as observed in the data, but with no attraction, it is possible to determine the bias of T cell movement towards the infection site in the absence of actual attraction, κ_a^0. We thus used this estimate for κ_a^0 in our test of whether the detected bias κ_a in actual data was significantly different from κ_a^0.

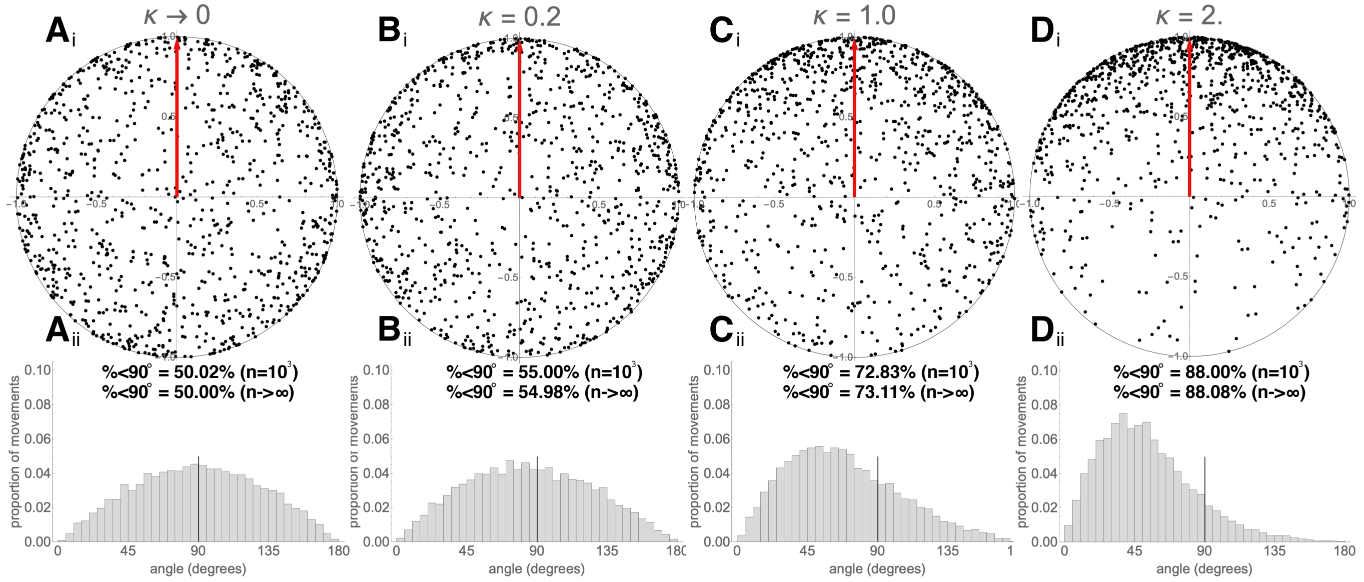

Figure S5: Illustration of the random vectors generated using the von Mises-Fisher (vMF) distribution. The vMF distribution generates direction vectors with preference toward a given direction, with the strength of preference determined by the concentration parameter κ. We sampled 1000 directions from a vMF distribution for several values of κ (noted on individual panels) and plotted those in a 2D image by removing the z-coordinates (panels i). We also show histograms of the actual angles between the movement vector and the vector to the parasite (panels ii, angle metric defined in Figure S3). In panels ii we also show the percents of acute angles generated for the $n = 1000$ pseudorandomly sampled vectors which were calculated using eqn. (S4).

How to create stochastic simulations based on the vMF distribution. We simulated a moving cell by generating a set of movements, each of which is defined by a distance traversed and a direction vector of the cell's movement. To generate the movement distances, we used Mathematica to estimate the best distribution to fit the distances from a real dataset, which resulted in the Pareto Type IV (generalized Pareto, GP) distribution:

$$f(x) = \left(1 + ((x - \mu)/k)^{(1/\gamma)}\right)^{-\alpha}, \tag{S8}$$

with the following best fit parameters: minimum value $k = 7$, location parameter $\mu = 0$, scale parameter $\alpha = 2.7$, and inequality parameter $\gamma = 0.68$, assuming that these movements occur in 2 min intervals. These parameters were found by fitting the GP distribution to movement data from datasets #4 and #5 pooled together, using the function `FindDistributionParameters` on a `ParetoDistribution` in Mathematica. Of note, we also found that the GP best fits the movements of malaria-specific CD8 T cells in the absence of a malaria infection [17].

To simulate attracted movement of T cells towards a parasite, each direction was chosen from the vMF distribution with the angle between T cell movement and the parasite given in eqn. (1).

The concentration κ_a was chosen based on the desired amount of attraction ($\kappa_a > 0$ corresponds to attraction, $\kappa_a = 0$ corresponds to no bias, and $\kappa_a < 0$ corresponds to repulsion). To create the desired vector, we first chose a vector with bias toward direction {0,0,1}, which simplifies the process to choosing x and y pseudorandomly from a normal distribution $N(0, 1)$ and choosing z based on the von Mises-Fisher distribution, using

$$z = 1 + (\ln(r) + \ln(1 + (1 - r)\frac{e^{-2\kappa_t}}{r}))/\kappa_t, \tag{S9}$$

where r is chosen uniformly between 0 and 1, exclusively [18]. Then we weighted x and y to place the chosen vector on the unit sphere, and used a rotation transform to adjust the generated vector with respect to the desired bias direction.

To simulate a correlated (persistent) random walk, each movement direction was chosen from a vMF distribution with a vector input of the vector from the cell's previous position to the cell's current position. The concentration κ_t was chosen based on the desired amount of movement persistence, with $\kappa_t > 0$ corresponding to persistence, $\kappa_t = 0$ corresponding to randomness, and $\kappa_a < 0$ corresponding to the cell turning back on itself. For a constant parasite position, a sequence of movements was simulated consecutively, resulting in a list of cell positions similar to those from the experimental datasets.

We also simulated a walk with both attraction to a parasite and persistence by choosing two directions with bias as described previously and summing each pair of directions to find a direction with bias influenced by both the parasite and movement persistence. This framework can easily be extended to more than two biases in a similar way.

How to create stochastic simulations based on the Ornstein-Uhlenbeck process. The Ornstein-Uhlenbeck (OU) process is a stochastic process that generates a correlated (persistent) random walk [5, 13]. The OU process chooses each position to move to using the following formula:

$$\overrightarrow{\mathbf{v_{n+1}}} = j\left(1 - \frac{dt}{p}\right)\overrightarrow{\mathbf{v_n}} + \frac{j\sqrt{dt^3}}{\sqrt{p}}\overrightarrow{\mathbf{c}}, \tag{S10}$$

where $\overrightarrow{\mathbf{v_n}}$ is the nth displacement vector of a cell (the vector from the nth position of the cell to the $n+1^{\text{th}}$ position of the cell) and $\overrightarrow{\mathbf{v_{n+1}}}$ is the $n+1^{\text{th}}$ displacement vector, j is the expected movement length (equivalent to the speed times dt), p is the persistence time, dt is the timestep in simulations, and $\overrightarrow{\mathbf{c}}$ is a pseudorandomly chosen vector to introduce randomness. In this model, p determines the timescale at which movements are correlated (i.e., the timescale of the persistence of the random walk). We adjusted the OU process to simulate cells with attraction to a specific point in space, rather than relative to the previous movement vector, using the following formula:

$$\overrightarrow{\mathbf{v_{n+1}}} = j\left(1 - \frac{dt}{p}\right)\overrightarrow{\mu_{\mathbf{n}}} + M\frac{j\sqrt{dt^3}}{\sqrt{p}}\overrightarrow{\mathbf{c}}, \tag{S11}$$

where $\overrightarrow{\mu_{\mathbf{n}}}$ is a vector from thethe n^{th} position of the cell to the parasite, M is a multiplier that increases the effect of the randomness, and other parameters are the same as the persistent OU model (eqn.

(S10)). In this model, the strength of attraction to the parasite is determined by p, with larger values determining stronger attraction, and M negates the impact of such attraction by introducing higher degree of randomness in the movements. For each analyses, we simulate cell's positions at a regular interval dt which is typically small (e.g., 1 second), and then we sample every 100th position to gain simulated data comparable to real cells (one position per 100 seconds). We typically chose the following parameters to simulate walks with attraction towards the parasite: $p = 1.1s$ to $10.1s$, $dt = 1s$, $j = 0.02\mu$m (resulting from a speed of 1.2μm$/s$), and $M = 500$. In these simulations, cells have a starting distance of 100μm.

The von Mises-Fisher distribution-based metric is the most powerful metric at detecting attraction from the tested alternatives. We performed two sets of stochastic simulations in order to determine which of our metrics is most powerful, i.e., which metric requires the least amount of data to detect attraction, or equivalently, which metric detects more/stronger attraction for the same amount of data. In the first set of simulations, we described movement lengths of T cells by the generalized Pareto distribution and described attraction towards the infection site by the vMF distribution with different concentration parameters. In the second set of simulations, we described the movement of T cells using the modified OU process with different persistence times (see eqn. (S11)). We found that independent of the method used to simulate attracted movement for the same number of movements (data points), the vMF distribution-based metric allowed us to detect the smallest bias in movement. Equivalently, for the same degree of attraction, the vMF distribution-based metric required the least data to detect attraction (Figure S7). This demonstrates the stronger sensitivity of the vMF-based metric. We also note that as κ_a approaches 0, the percent of cells detected as attracted decreases, indicating a small false positive rate.

References

1. McNamara, H. A. *et al.* Up-regulation of LFA-1 allows liver-resident memory T cells to patrol and remain in the hepatic sinusoids. *Science Immunology,* 1–10 (2 2017).
2. Kelemen, R. K., Rajakaruna, H., Cockburn, I. A. & Ganusov, V. V. Clustering of activated CD8 T cells around malaria-infected hepatocytes is rapid and is driven by antigen-specific cells. *Frontiers in immunology* **10,** 2153. ISSN: 1664-3224 (2019).
3. Turchin, P. Quantitative Analysis of Movement: Measuring and Modeling Population Redistribution in Animals and Plants. 381. ISSN: 0033-5770 (1998).
4. Kelemen, R. K. *et al.* Classification of T Cell Movement Tracks Allows for Prediction of Cell Function. *International Journal of Computational Biology and Drug Design* (2014).
5. Ariotti, S. *et al.* Subtle CXCR3-Dependent Chemotaxis of CTLs within Infected Tissue Allows Efficient Target Localization. eng. *Journal of immunology (Baltimore, Md. : 1950)* **195.** ISSN: 1550-6606 (Dec. 2015).
6. Duchesne, T., Fortin, D. & Rivest, L.-P. Equivalence between Step Selection Functions and Biased Correlated Random Walks for Statistical Inference on Animal Movement. *PloS one* **10,** e0122947. ISSN: 1932-6203 (4 2015). epublish.
7. Mardia, K. V. *Directional statistics* Wiley; 2000.
8. Fisher, N. I. *Statistical analysis of spherical data* eng. Cambridge University Press, 1987.

9. Pawitan, Y. *In All Likelihood: Statistical Modelling and Inference Using Likelihood* 544 (Oxford University Press, 2001).

10. Moses, M. E., Cannon, J. L., Gordon, D. M. & Forrest, S. Distributed Adaptive Search in T Cells: Lessons From Ants. *Frontiers in Immunology* **10,** 1357. ISSN: 1664-3224 (2019). epublish.

11. Ahmed, D. A. & Petrovskii, S. V. Analysing the impact of trap shape and movement behaviour of ground-dwelling arthropods on trap efficiency. *Methods Ecol Evol* **10,** 1246–1264. ISSN: 2041-210X (2019).

12. Uhlenbeck, G. E. & Ornstein, L. S. On the Theory of the Brownian Motion. *Phys. Rev.* **36,** 823–841. `https://link.aps.org/doi/10.1103/PhysRev.36.823` (5 Sept. 1930).

13. Wu, P.-H., Giri, A., Sun, S. X. & Wirtz, D. Three-dimensional cell migration does not follow a random walk. *Proceedings of the National Academy of Sciences* **111,** 3949–3954. ISSN: 0027-8424. eprint: `https://www.pnas.org/content/111/11/3949.full.pdf`. `https://www.pnas.org/content/111/11/3949` (2014).

14. Jerison, E. R. & Quake, S. R. Heterogeneous T cell motility behaviors emerge from a coupling between speed and turning in vivo. *eLife.* `https://elifesciences.org/articles/53933` (2020).

15. Beltman, J. B., Marée, A. F. M. & de Boer, R. J. Analysing immune cell migration. *Nat Rev Immunol* **9,** 789–798 (2009).

16. Cockburn, I. A. *et al.* In vivo imaging of CD8+ T cell-mediated elimination of malaria liver stages. eng. *Proc Natl Acad Sci U S A* **110,** 9090–9095. `http://dx.doi.org/10.1073/pnas.1303858110` (May 2013).

17. Rajakaruna, H., O'Connor, J., Cockburn, I. A. & Ganusov, V. V. *Environment-imposed constraints make Brownian walkers efficient searchers* BioRxiv. 2020. `https://doi.org/10.1101/2020.11.06.371690`.

18. *Vectors with a certain magnitude in Mathematica* `https://mathematica.stackexchange.com/questions/13038/vectors-with-a-certain-magnitude-in-mathematica/13042#13042`.

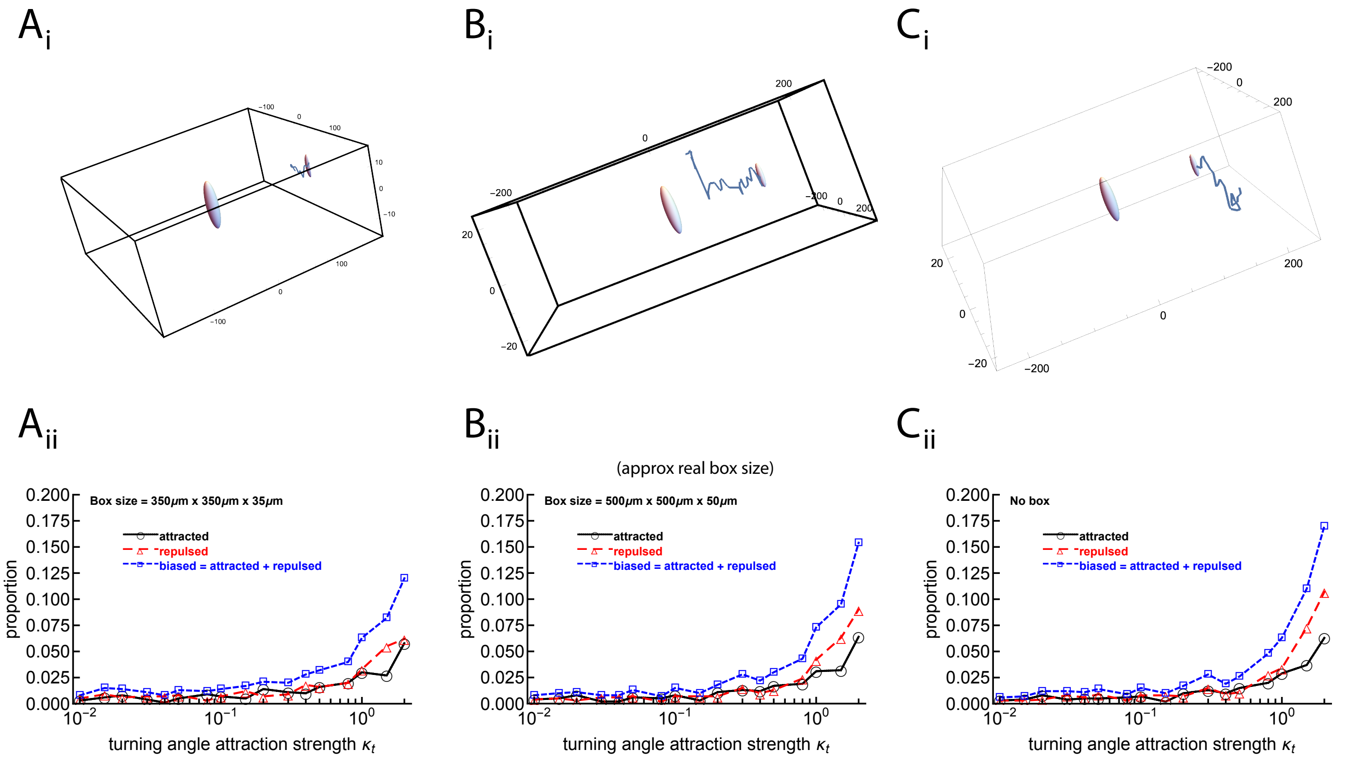

Figure S6: We quantified bias in the angle distribution (vMF distribution-based) metric and found that the number of cells detected as repulsed depends on the size of the imaging volume when the cells are persistent. We run stochastic simulations of T cells moving in a small "boxed" space ($350 \times 350 \times 35$ μm, panels A), a regular boxed space as used in intravital imaging experiments ($500 \times 500 \times 50$ μm, panels B), and in open space (panels C). The parasite is assumed to be located in the center of the box and T cells start movement at 150 μm from the parasite at coordinates $(x, y, z) = (150, 0, 0)$. We assume 50 movements per simulated T cell, with movement lengths described by the Generalized Pareto distribution (eqn. (S8)) with parameters given in Materials and Methods. To simulate a persistent random walk, we assume that T cell turning angles are described by a vMF distribution with constant κ_t determining the bias of a T cell walk (see Materials and Methods for more detail). A cell is assumed to be out of the imaging volume when the cell's position is outside of the defined imaging box (in A and B) which then results in fewer than 50 moves recorded. For each cell's track, we determine if the cell is attracted ($\kappa_a > 0$), repulsed ($\kappa_a < 0$), or unbiased ($\kappa_a = 0$) to the parasite's position by comparing the distribution of angles to the parasite to a vMF distribution (Figure S3) and estimating the concentration κ_a. For each concentration determining the persistence of the random walk κ_t, we simulate movement of 1000 cells. In Panels i are examples of a cell whose path is cut off by the small box (Ai), a cell detected as attracted (Bi), or a cell detected as repulsed (Ci). Note that in Panels i the figures are not to scale, resulting in the spheres representing the cells being elongated. In these examples, the parasite and cell's starting position and the cell's track are shown. Parasites are depicted as a sphere with a radius of 40 μm representing the infected hepatocyte [2, 16].

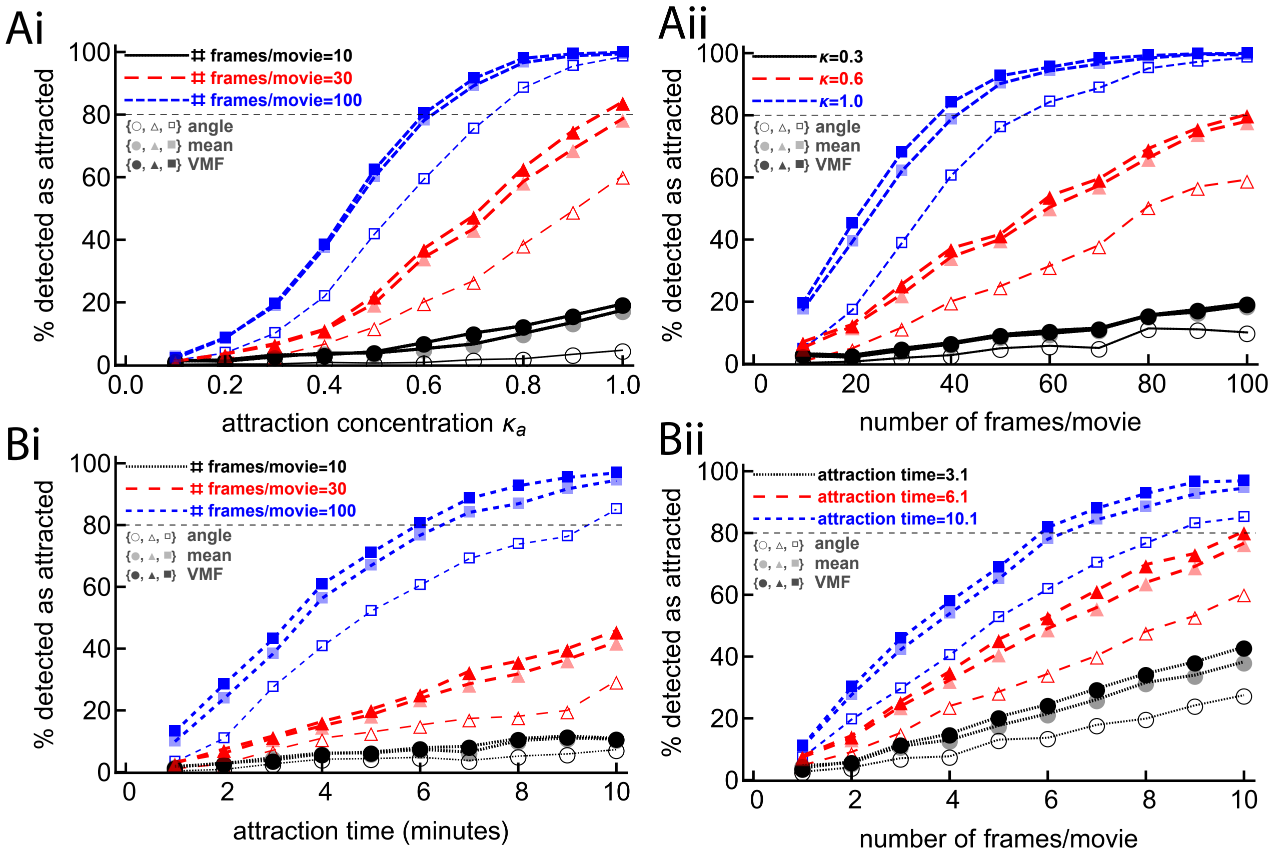

Figure S7: The third metric, based on the vMF distribution, is the most powerful out of the tested metrics (demonstrated by its percent of cells detected as attracted always being higher than the other metrics). We simulated T cell movement with varying degrees of bias towards the parasite for different movement durations using two different frameworks: using vMF distribution-based simulations (panels A and Figure 4) or using the Ornstein-Uhlenbeck (OU) process (panels B and eqn. (S11)). For each simulated trajectory we determined the percent of cells detected as attracted using the angle metric (metric 1, open symbols), the vMF distribution-based metric (metric 3, solid symbols), and the mean angle metric (metric 4, transparent symbols). The distance metric (metric 2) is not shown to avoid clutter and because it performs similarly to metric 1. Cells were detected as attracted if their movement was statistically different from unbiased. In total, we simulated 1000 cells per every set of parameters (see Additional experimental details section for exact values of parameters used in simulations).

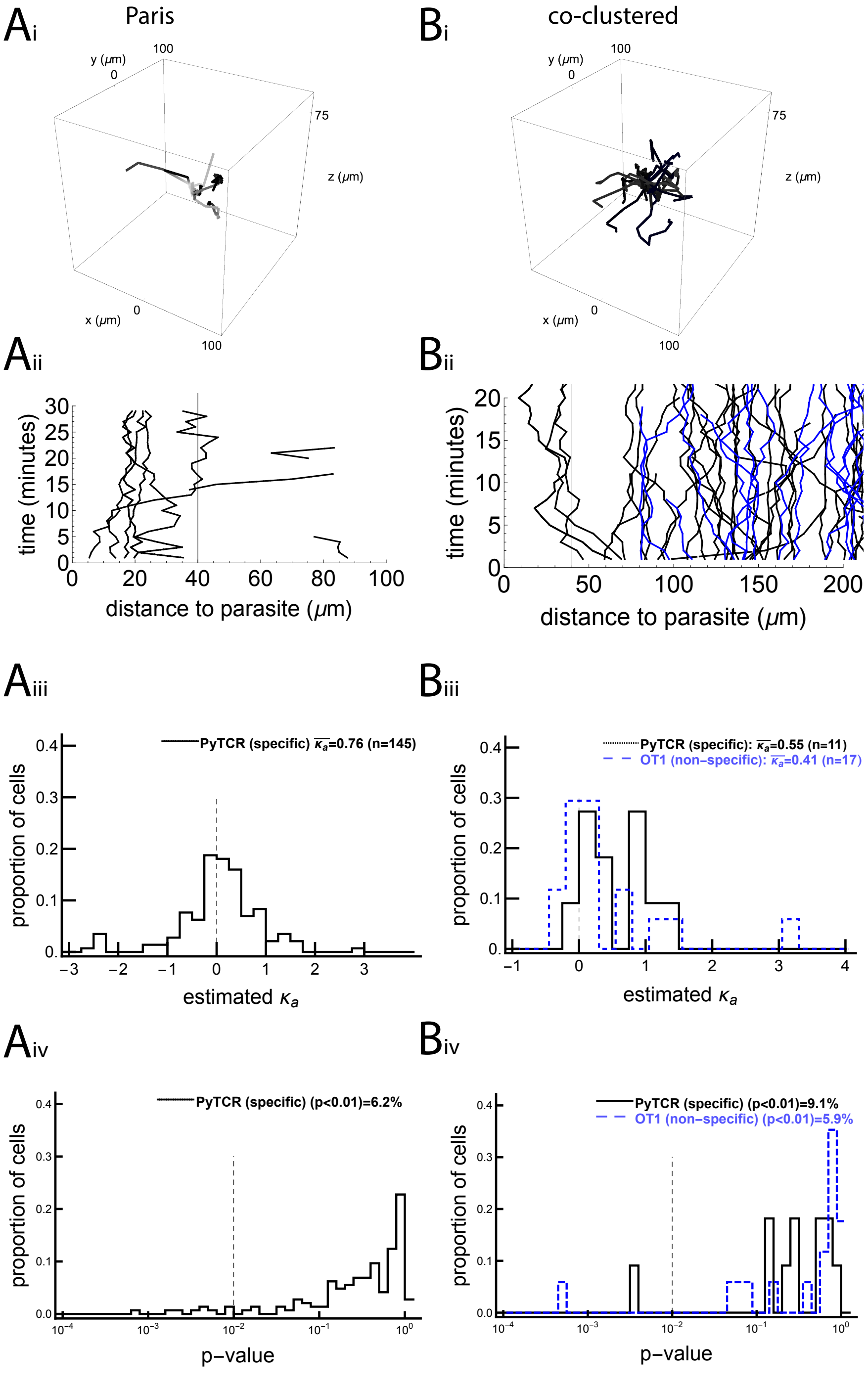

Figure S8: A minority of activated CD8 T cells display movement bias to the liver stage. This analysis was similar to Figure 1, for the previously published ("Paris" and "co-clustered") data [4, 16]. In the Paris data there were no nonspecific cells, but there were four mice with several infection sites each. In the co-clustered data, we imaged CD8 T cells, specific to Py (PyTCR) or specific to a irrelevant antigen (OT1), in one mouse with one Py liver stage. For the Paris data, we detected 5 PyTCR cells as attracted and 4 PyTCR cells as repulsed. For the co-clustered data, we detected 1 PyTCR cells and 1 OT1 cells as attracted and 0 PyTCR cells and 0 OT1 cells as repulsed.

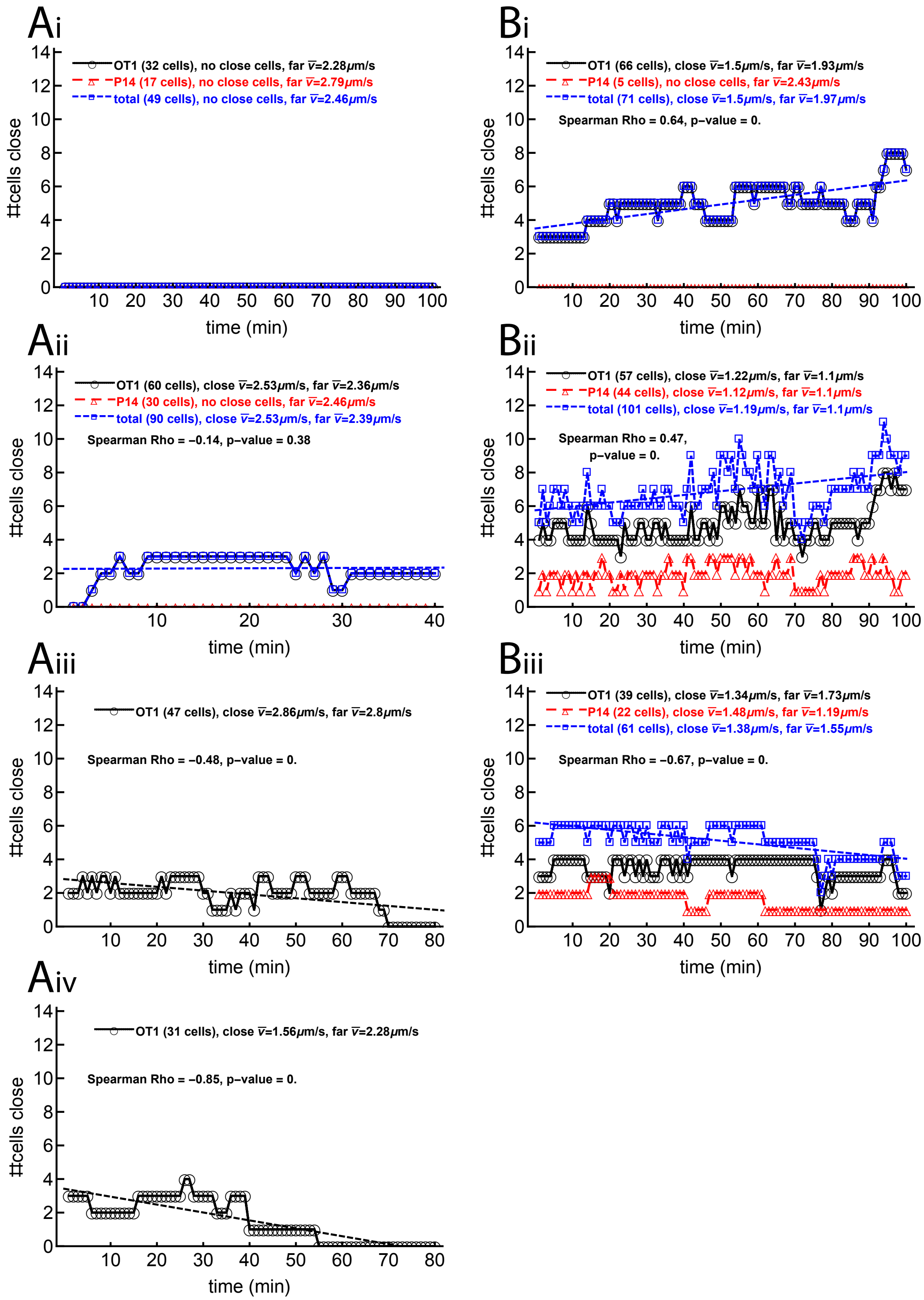

Figure S9: The numbers of cells close to the parasite change more over time for large clustered datasets than for unclustered/small clustered datasets, according to the Spearman correlation test. The size of the cluster (the number of cells close to a parasite) is of interest, because if the cluster size increases, then cells are clearly coming to the parasite and not leaving. Here we show the number of cells close to the parasite (within 40μm from the parasite) at each time point, with one panel for each parasite. The left column is for the unclustered/small clustered dataset, and the right column is for the large clustered dataset.

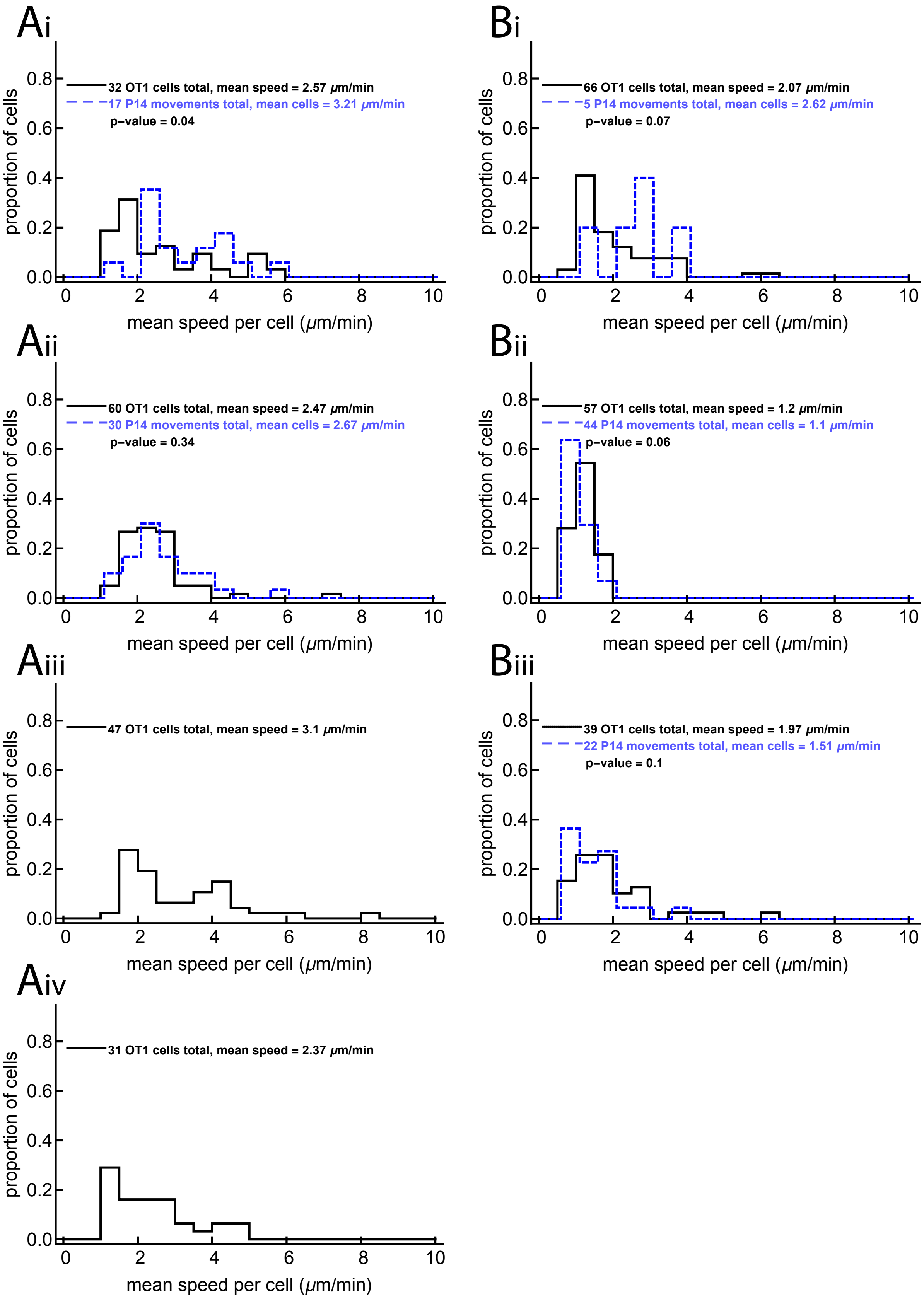

Figure S10: Distribution of average speed per cell in different imaging experiments. Left panels are for the unclustered/small clustered dataset, and right panels are for the large clustered dataset. The p-values come from the Mann Whitney test.

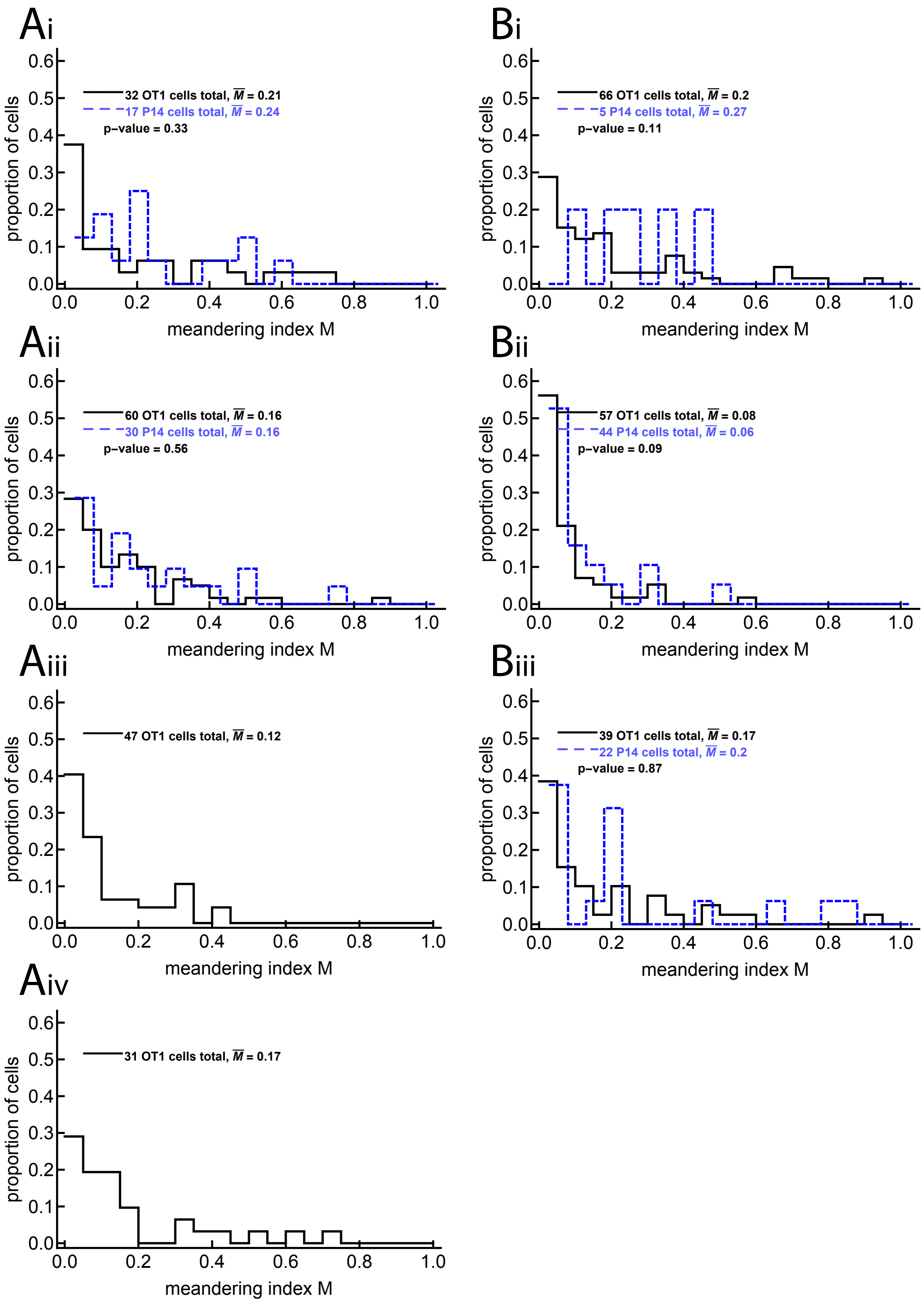

Figure S11: We display the distributions of meandering indices for cells in the experiments with dataset #1 (unclustered/small clustered dataset) and #2 (large cluster dataset). The meandering index M is calculated as the distance between the first and last recorded positions of the cell divided by the total length of the cell's movement in space; it is therefore between 0 and 1, with 0 equivalent to the cell returning to its original position and 1 meaning the cell moved in a straight line. In our real data, the meandering indices are mostly around 0.15 to 0.2, and are identical between corresponding specific and non-specific datasets. The p-values come from a Mann Whitney test.

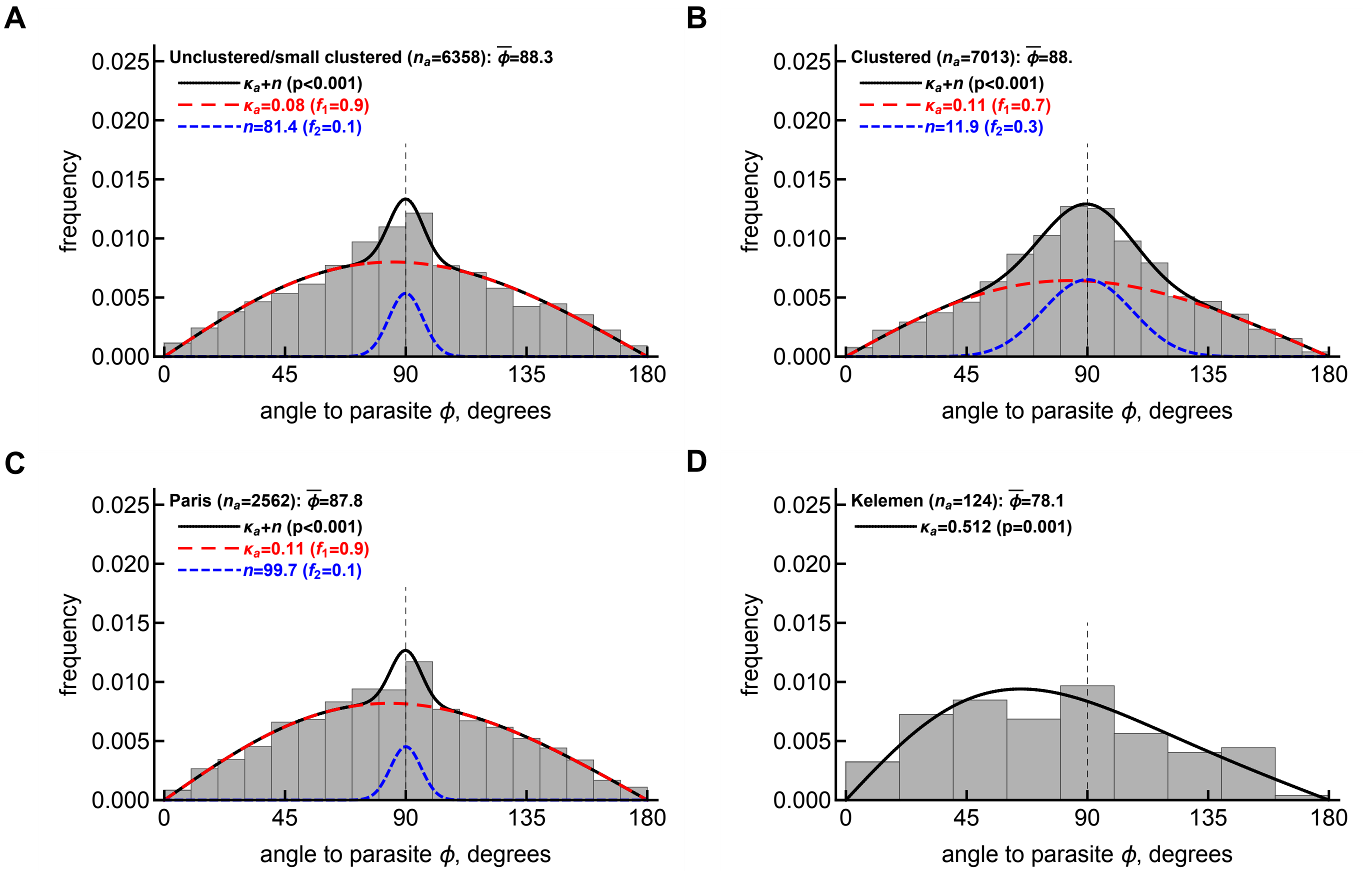

Figure S12: The von Mises-Fisher distribution describes relatively well the distributions of angles to the parasites from four datasets. We show the distributions of angles to the parasite of *Plasmodium*-specific liver-resident CD8 T cells, as outlined in Materials and Methods, for the unclustered/small clustered dataset (A), clustered dataset (B), Paris dataset (C), and co-clustered dataset (D). We fit either a vMF distribution (given in eqn. (1)) or a mixture of distributions (eqn. (S6)) to these data and estimated the concentration parameter κ_a (noted on individual panels). Fits of the model are shown by lines. We compared every fit with the fit of a simpler model (a single vMF distribution fit in panels A-B or a null distribution of angles with $\kappa_a \to 0$ in panel D) using a likelihood ratio test; resulting p-values are shown on individual panels. For each dataset we also plot the total number of angles in the dataset (n_a) and the average angle to the parasite ($\bar{\phi}$).

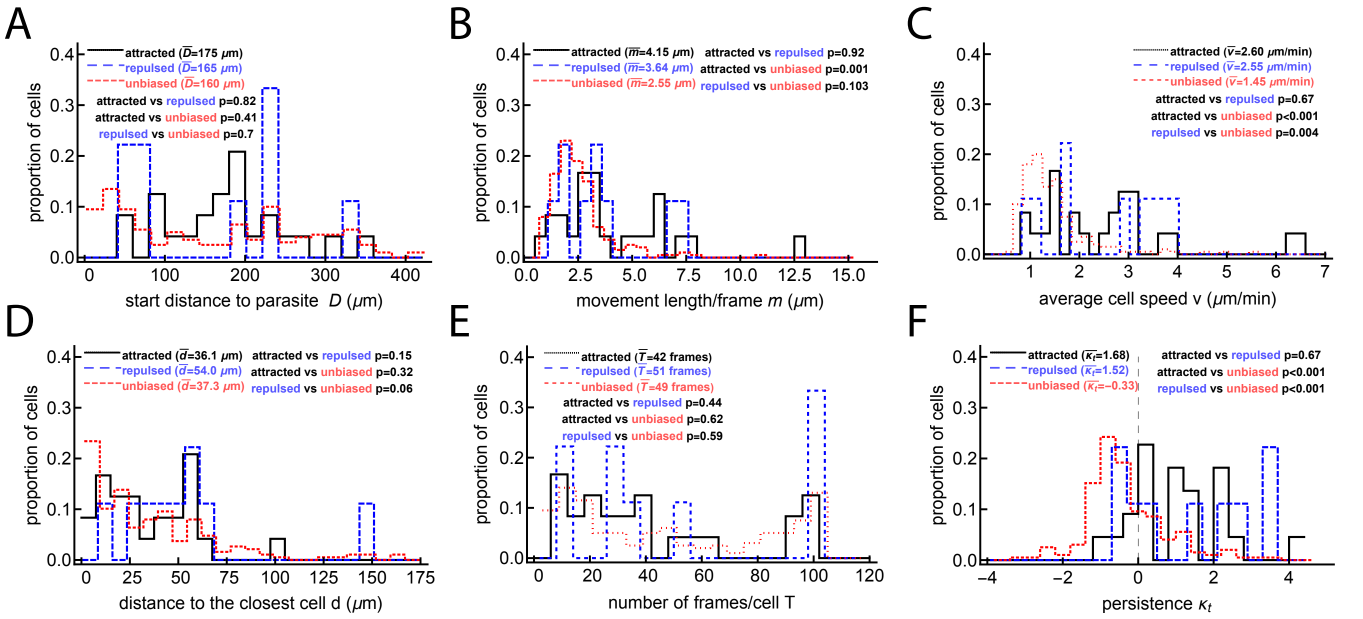

Figure S13: Speed and turning angle both correlate with detection of attraction when several T cells have found the parasite. These are metadata results from tests that use data divided into attracted, repulsed, and unbiased T cells for the large clustered dataset (dataset #2). For more details, see Figure 2.

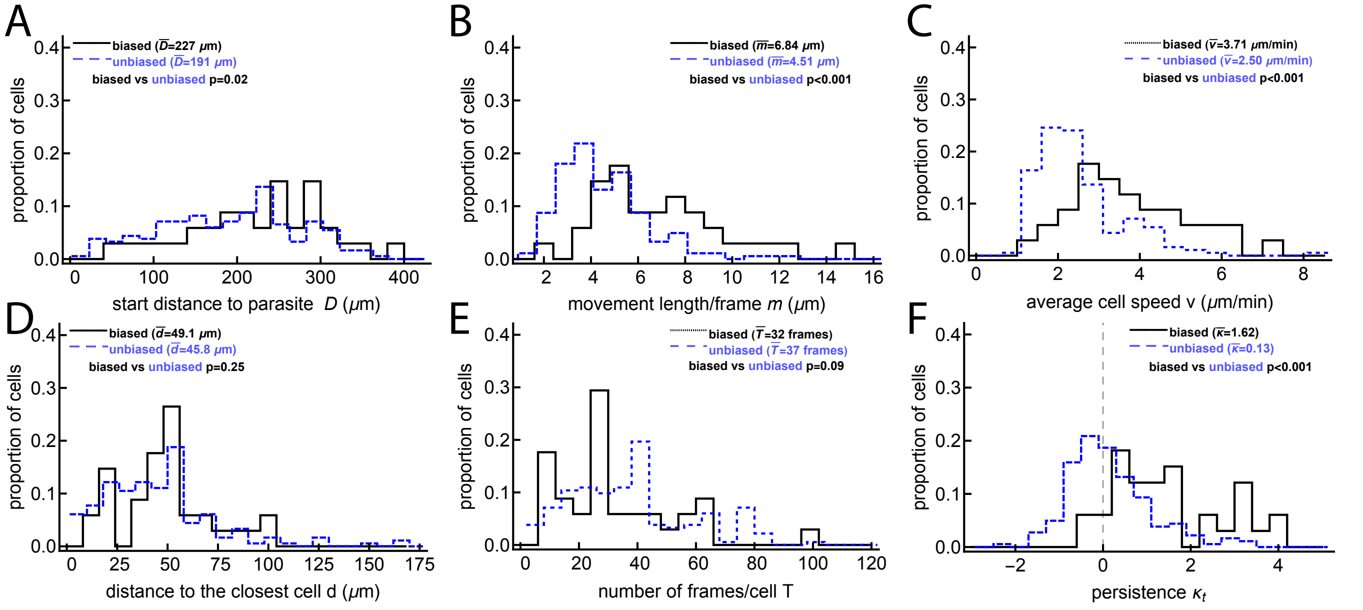

Figure S14: Speed and turning angle both correlate with detection of attraction when no or few T cells have found the parasite. These are metadata results from tests that use data divided into biased and unbiased T cells for the unclustered/small clustered dataset (dataset #1). For more details, see Figure 2.

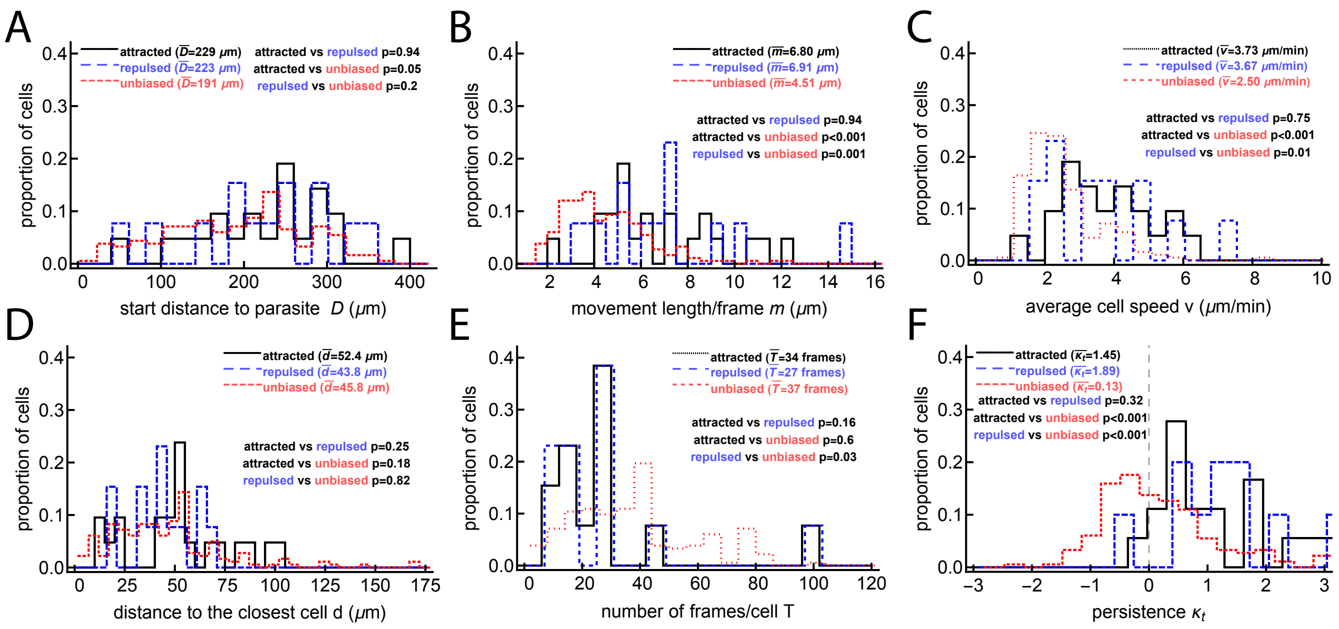

Figure S15: Speed and turning angle both correlate with detection of attraction when no or few T cells have found the parasite. These are metadata results from tests that use data divided into attracted, repulsed, and unbiased T cells for the unclustered/small clustered dataset. For more details, see Figure 2.

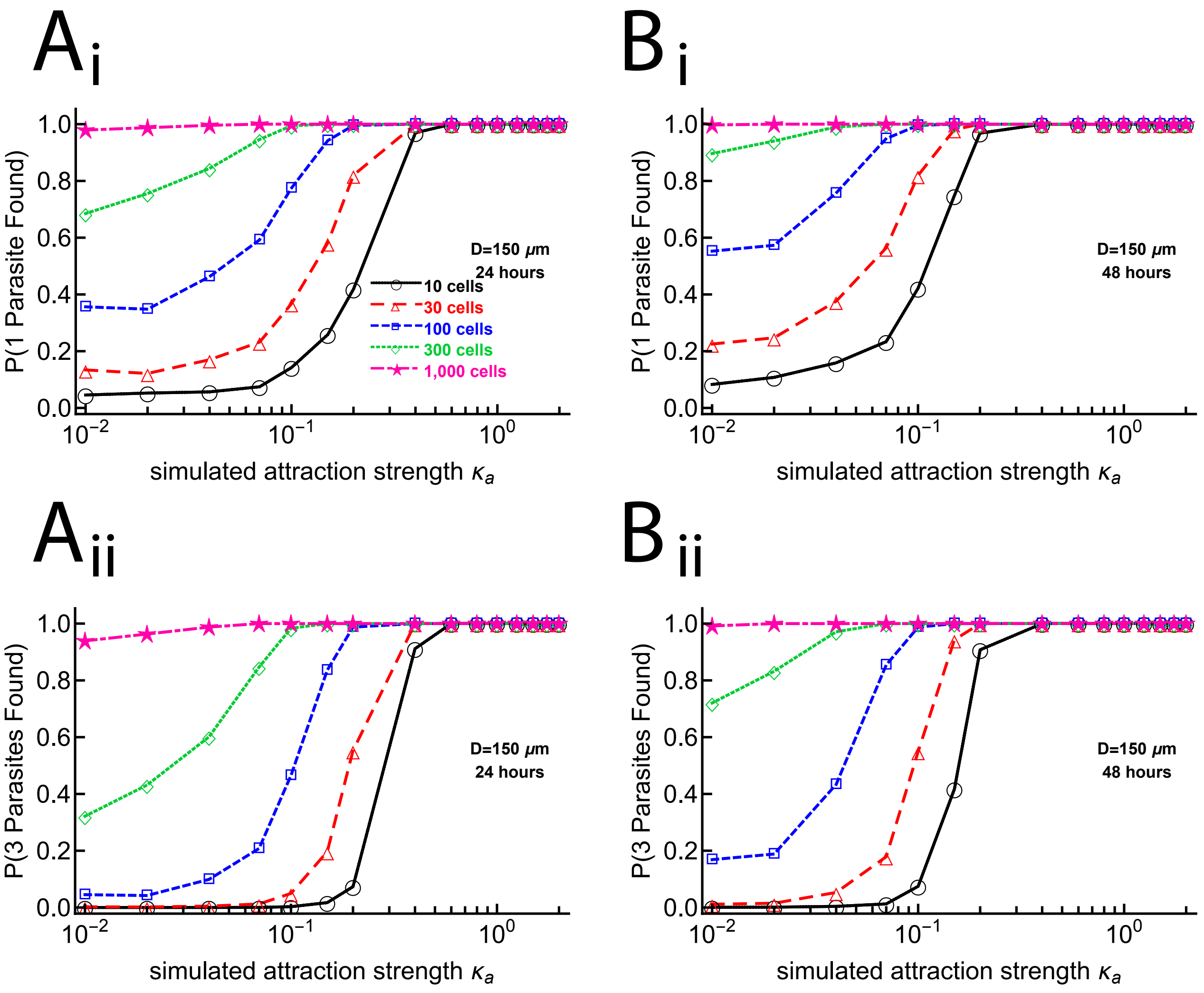

Figure S16: Many CD8 T cells per parasite are needed to ensure that all parasites are found within 48 hours after infection. Groups of 10, 30, 100, 300, and 1000 cells per parasite are simulated 1000 times for values of κ_a between 0 and 2 with a starting distances of 150 μm and are "sampled" every two minutes for lengths of 24 (panels A) or 48 (panels B) hours. Each parasite that has a cell reach within 40μm of it is considered a success. Panels i show the probability of a parasite being found, while panels ii show the probabilities of three parasites being found. If there are 100 cells per parasite, a κ_a of 1.0 is required for essentially all parasites to be found within 48 hours.
